# Microbial metabolite drives ageing-related clonal haematopoiesis via ALPK1

**DOI:** 10.1038/s41586-025-08938-8

**Published:** 2025-04-23

**Authors:** Puneet Agarwal, Avery Sampson, Kathleen Hueneman, Kwangmin Choi, Niels Asger Jakobsen, Emma Uible, Chiharu Ishikawa, Jennifer Yeung, Lyndsey Bolanos, Xueheng Zhao, Kenneth D. Setchell, David B. Haslam, Jessica Galloway-Pena, John C. Byrd, Paresh Vyas, Daniel T. Starczynowski

**Affiliations:** 1 Division of Experimental Hematology and Cancer Biology, Cincinnati Children’s Hospital Medical Center, Cincinnati, OH USA; 2 MRC Molecular Haematology Unit, Oxford Centre for Haematology, Weatherall Institute of Molecular Medicine, Radcliffe Department of Medicine, University of Oxford, Oxford, UK; 3 Division of Pathology and Laboratory Medicine, Cincinnati Children’s Hospital Medical Center, Cincinnati, OH USA; 4 Division of Infectious Diseases, Cincinnati Children’s Hospital Medical Center, Cincinnati, OH USA; 5 Department of Pediatrics, University of Cincinnati College of Medicine, Cincinnati, OH USA; 6 Department of Veterinary Pathobiology, Texas A&M University, College Station, TX USA; 7 Division of Hematology, Department of Internal Medicine, University of Cincinnati, Cincinnati, OH USA; 8 University of Cincinnati Cancer Center, Cincinnati, OH USA; 9 Department of Cancer Biology, University of Cincinnati College of Medicine, Cincinnati, OH USA

**Keywords:** Cell signalling, Haematological diseases

## Abstract

Clonal haematopoiesis of indeterminate potential (CHIP) involves the gradual expansion of mutant pre-leukaemic haematopoietic cells, which increases with age and confers a risk for multiple diseases, including leukaemia and immune-related conditions^1^. Although the absolute risk of leukaemic transformation in individuals with CHIP is very low, the strongest predictor of progression is the accumulation of mutant haematopoietic cells^2^. Despite the known associations between CHIP and increased all-cause mortality, our understanding of environmental and regulatory factors that underlie this process during ageing remains rudimentary. Here we show that intestinal alterations, which can occur with age, lead to systemic dissemination of a microbial metabolite that promotes pre-leukaemic cell expansion. Specifically, ADP-d-glycero-β-d-manno-heptose (ADP-heptose), a biosynthetic bi-product specific to Gram-negative bacteria^3–5^, is uniquely found in the circulation of older individuals and favours the expansion of pre-leukaemic cells. ADP-heptose is also associated with increased inflammation and cardiovascular risk in CHIP. Mechanistically, ADP-heptose binds to its receptor, ALPK1, triggering transcriptional reprogramming and NF-κB activation that endows pre-leukaemic cells with a competitive advantage due to excessive clonal proliferation. Collectively, we identify that the accumulation of ADP-heptose represents a direct link between ageing and expansion of rare pre-leukaemic cells, suggesting that the ADP-heptose–ALPK1 axis is a promising therapeutic target to prevent progression of CHIP to overt leukaemia and immune-related conditions.

## Abstract (web summary)

ADP-heptose binds to ALPK1, triggering transcriptional reprogramming and NF-κB activation, endowing pre-leukaemic cells with a competitive advantage due to excessive clonal proliferation.

## Main

CHIP arises in older individuals due to leukaemia-associated mutations, primarily in epigenetic modifiers *DNMT3A*, *TET2* and *ASXL1*, within haematopoietic stem cells (HSCs), leading to the emergence of pre-leukaemic cells. While individuals with CHIP maintain normal blood counts, they face elevated risks of haematologic cancers and cardiopulmonary diseases^6^. Although the absolute risk of leukaemic transformation is low, mutant haematopoietic cell burden predicts progression to myelodysplastic syndromes (MDS), acute myeloid leukaemia (AML) and immune-related conditions^7^. Individuals with CHIP can either have a static or expanding pool of mutant haematopoietic cells^8^, with larger clone sizes correlating with a higher risk of myeloid malignancies and immune-related conditions, such as coronary heart disease and rheumatoid arthritis^9^. Moreover, autoimmune and inflammatory disorders are implicated in pre-leukaemic cell expansion and myeloid malignancy development^10–15^. Despite the link between ageing and myeloid malignancies, the signals driving pre-leukaemic cell expansion remain unclear. In recent studies, a germline variant leading to aberrant TCL1A activation was shown to promote pre-leukaemic cell expansion in CHIP with TET2 or ASXL1 mutations but not with DNMT3A mutations^16^. *DNMT3A*-mutant CHIP carries a fourfold increased risk of myeloid neoplasm^6^, yet factors contributing to the expansion of *DNMT3A*-mutant cells remain unidentified. Recent studies show that inactivating mutations in DNMT3A are enriched in older individuals and those with chronic intestinal inflammatory disorders^17,18^. We therefore investigated the effects of ageing-associated intestinal barrier dysfunction on the expansion of *DNMT3A*-mutant pre-leukaemic cells. Here we demonstrate that ADP-heptose, a biosynthetic by-product of Gram-negative bacteria, circulates in the blood of older individuals. ADP-heptose, a bacterial intermediary sugar in the biosynthesis of lipopolysaccharide (LPS), binds to the cytosolic receptor ALPK1 to trigger immune responses^3,4,19^. However, we found that ageing-associated circulating ADP-heptose directly induces the expansion of pre-leukaemic cells through ALPK1.

## Gut dysfunction drives pre-leukaemia

**Fig. 1:**
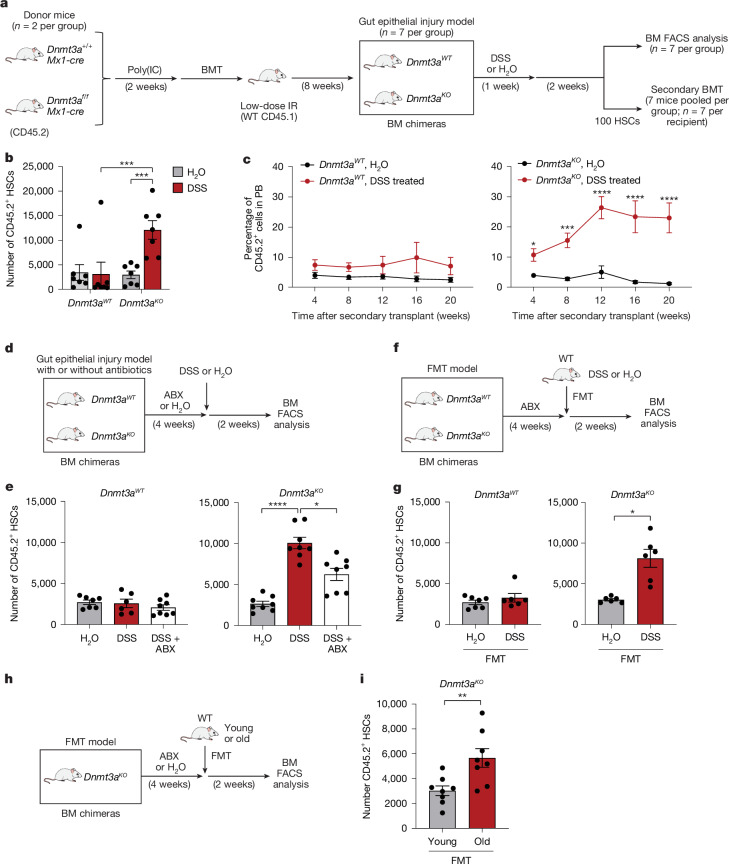
Intestinal epithelial injury and microbiome alterations contribute to pre-leukaemic HSC expansion. **a**, BM cells from mice (*n* = 2 mice per group) treated with polyinosinic:polycytidylic acid (poly(I:C)) were transplanted into wild-type (WT) mice conditioned at 2.5 Gy (*n* = 14 mice per group). Chimeric mice (*n* = 7 mice per group) were treated with water (H_2_O) or DSS (2.5%). Secondary transplants were performed with FACS-purified HSCs (CD45.2^+^Lin^−^KIT^+^SCA1^+^CD150^+^CD48^−^). **b**, The number of donor HSCs in the BM. **c**, Summary of donor-derived PB proportions. *n* = 8 or 10 mice per group. **d**, The experimental design to examine role of gut microbiota (adapted from **a**). Chimeric mice were pretreated with broad-spectrum antibiotics (ABX) for 4 weeks and then treated with DSS (*n* = 8 mice per group). Control mice were treated with H_2_O (*n* = 7 and 8 mice per group) or DSS (*n* = 6 and 8 mice per group). **e**, The number of donor HSCs in the BM. **f**, The experimental design to examine the contribution of microbiome using faecal microbiota transplantation (FMT; adapted from **a**). Chimeric mice were pretreated with antibiotics and then colonized with faecal material from H_2_O- or DSS-treated WT mice. *n* = 6 mice per group. **g**, The number of donor HSCs in the BM. **h**, Chimeric mice were pretreated with antibiotics and then colonized with faecal material from young (6–10 weeks) or old (>52 weeks) WT mice. *n* = 8 mice per group. **i**, The number of donor HSCs in the BM. Data are mean ± s.e.m. *P* values were calculated using two-tailed unpaired Student’s *t*-tests (**b**,**g**,**i**) and two-way analysis of variance (ANOVA) (**c**,**e**). *P < 0.05, **P < 0.01, ***P < 0.001, ****P < 0.0001. Data are representative of at least three independent experiments. FACS-gating schemes are shown in Supplementary Fig. 2. Source data

**Extended Data Fig. 1:**
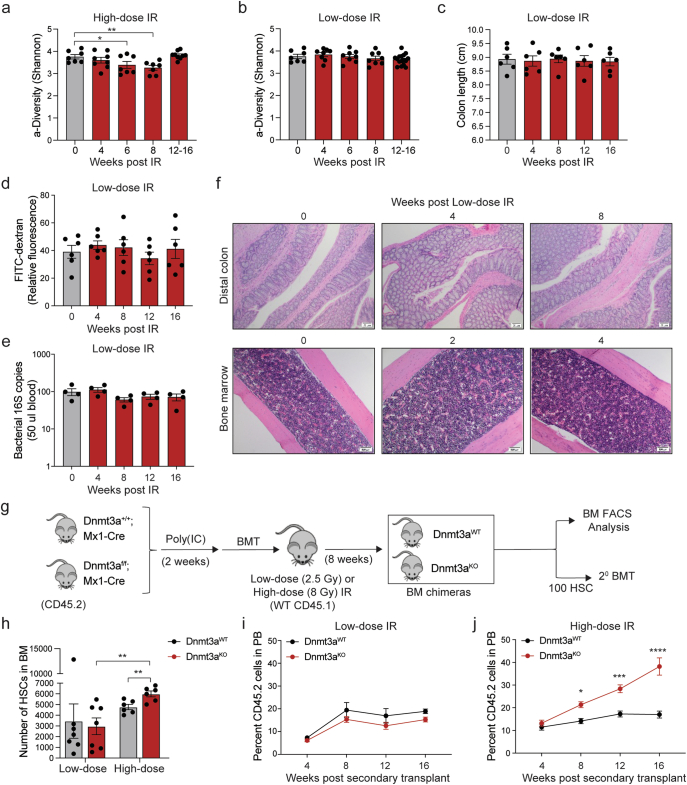
Loss of intestinal epithelial integrity contributes to pre-leukaemic HSC expansion. **a,b**, The gut microbiota of high-dose (8 Gy) (a) and low-dose (2.5 Gy) (b) irradiated mice were analysed for alpha-diversity, including observed operational taxonomic units (OTUs) and Shannon indices. **c**, Colon length in low-dose irradiated mice (n = 6 mice per time point). **d**, Intestinal permeability of low-dose irradiated mice measured by FITC-dextran translocation to the plasma following oral gavage (*n* = 6 mice per time point). **e**, Bacterial 16S gene copies measured in PB of low-dose irradiated mice (*n* = 4 mice per time point). **f**, Histological staining of distal colons (upper panel) and BM (lower panel) at the indicated weeks post-irradiation. Scale bars: 100 μm. Data are representative of 4 independent experiments. **g**, Experimental design to examine the effect of irradiation. Chimeric mice were generated by irradiating the recipient mice at either high-dose (8 Gy) or low-dose (2.5 Gy), and flow cytometry was performed on BM. Secondary transplants were performed with purified donor HSCs (CD45.2+). **h**, Number of donor HSCs (Lin-cKit+Sca1 + CD150 + CD48-) in the BM. Error bars represent the SEM. (*n* = 7 low-dose irradiated mice per group; *n* = 6 high-dose irradiated mice per group). **i,j**, Donor-derived proportions in the PB of recipient mice transplanted with low-dose irradiated (i), and high-dose irradiated mice (j) (*n* = 11 mice per group). Error bars represent the SEM. *P* values were calculated for the indicated comparisons using a two-tailed unpaired Student’s t-test (a and h) and a two-way ANOVA (b, c, d, e, i, and j). Source data

**Extended Data Fig. 2:**
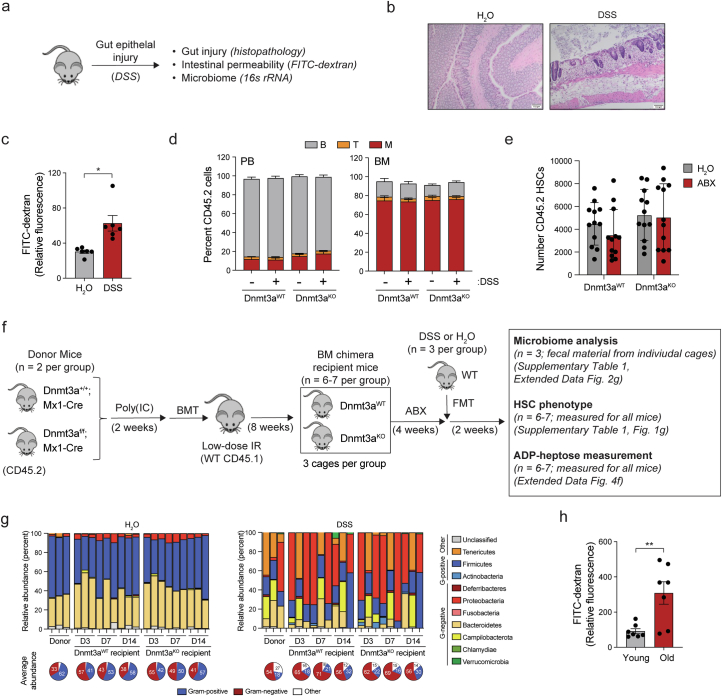
Microbiome alterations due to intestinal injury contribute to pre-leukaemic cell expansion. **a**, Experimental design. (adapted from Fig. 1a). **b**, Histological staining of distal colon tissues day 7 post-treatment with DSS. Scale bars: 100 μm. Data are representative of 6 independent experiments. **c**, Intestinal permeability was measured by FITC-dextran translocation to the plasma following oral gavage in H_2_O- or DSS-treated WT mice for 7 days (*n* = 6 mice per group). **d**, Myeloid (Gr1+ CD11b+) and lymphoid (B220+ CD19+ or CD3+) donor-derived proportions in the PB and BM at 16 weeks (*n* = 6 mice per group). **e**, Number of donor HSCs (Lin-cKit+Sca1+ CD150+ CD48−) in the BM of mice engrafted with Dnmt3a^WT^ or Dnmt3a^KO^ BM cells and then treated with broad-spectrum antibiotics (*n* = 12 mice per group). **f**, Outline of experimental design. **g**, Taxonomic composition of major bacterial phyla in faecal homogenate samples of indicated recipient mice following colonization with faecal material from either H_2_O- or DSS-treated WT mice (*n* = 3 mice per group). Shown below is a summary of the average relative abundance of gram-negative (G-negative) and gram-positive (G-positive) bacterial taxa (*n* = 3 biological replicates per pie chart). Refer to Supplementary Table 1 for complete analysis. **h**, Intestinal permeability measured by FITC-dextran translocation to the plasma following oral gavage in young (6–10 weeks) or old (>52 weeks) mice (*n* = 7 mice per group). *P* values were calculated for the indicated comparisons using a two-tailed unpaired Student’s t-test (c, e, and h) and a two-way ANOVA (d). Source data

*DNMT3A*-mutant pre-leukaemic cells in CHIP generally exhibit slow growth but can expand rapidly on ageing^2,20^. A hallmark of ageing is disruption of gut homeostasis, marked by increased intestinal permeability and microbial dysbiosis^21^. Loss of DNMT3A expression or function, due to truncating or inactivating mutations, results in the expansion of HSCs in older humans and mice^22^. To examine the effects of intestinal barrier dysfunction on *DNMT3A*-mutant haematopoietic cells, we first assessed the competitive advantage of DNMT3A-deficient (*Dnmt3a*^*−/−*^) haematopoietic cells in mice exposed to a level of radiation that either damaged (high dose, 8 Gy) or spared (low dose, 2.5 Gy) the intestinal epithelial barrier (Extended Data Fig. 1a–f). As reported previously^23^, engraftment of *Dnmt3a*^*−/−*^ bone marrow (BM) cells into recipient mice conditioned with high-dose radiation resulted in an expansion of HSCs and a significant increase in peripheral blood (PB) chimerism (Extended Data Fig. 1g–j), but this expansion was not observed in the low-dose-conditioned mice (Extended Data Fig. 1g–i), suggesting that intestinal epithelial injury promotes *Dnmt3a*-mutant HSC expansion. To directly examine the consequences of intestinal epithelial injury on mutant HSCs, *Dnmt3a*^*−/−*^ BM cells were engrafted into low-dose-irradiated mice and then treated with dextran sulfate sodium (DSS) (Fig. 1a), which damages the intestinal epithelium, mimicking human ulcerative colitis, a common form of inflammatory bowel disorder (IBD)^24^ (Extended Data Fig. 2a–c). DSS induced significant expansion of *Dnmt3a*^*−/−*^ HSCs in BM and secondary recipient mice (Fig. 1b,c), coinciding with multilineage differentiation (Extended Data Fig. 2d). The expansion of *Dnmt3a*^*−/−*^ HSCs after intestinal epithelial injury was significantly reduced with broad-spectrum antibiotics (Fig. 1d,e). The antibiotics did not alter wild-type (WT) or *Dnmt3a*^*−/−*^ HSCs in the absence of intestinal epithelial injury, suggesting that microbial dysbiosis contributes to the expansion of *Dnmt3a*^*−/−*^ HSCs (Extended Data Fig. 2e).

**Extended Data Fig. 3:**
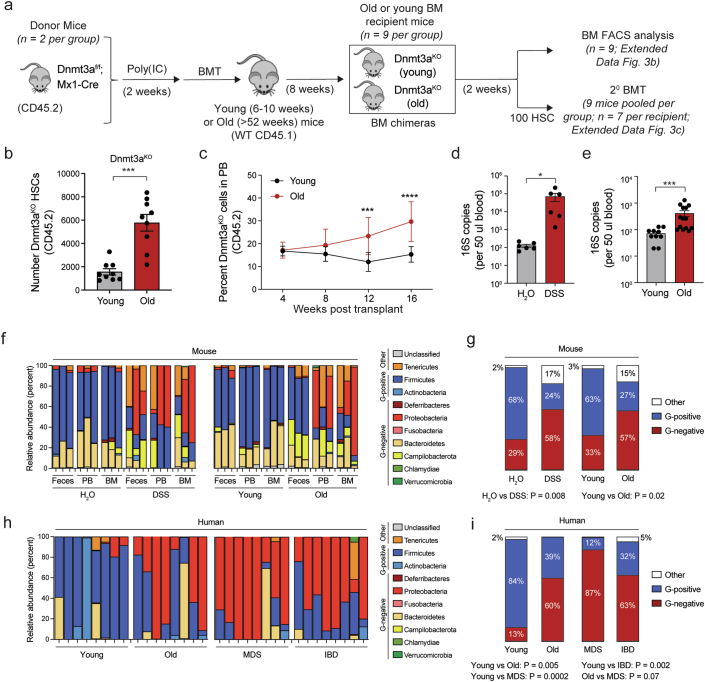
Evaluation of intestinal dysbiosis and aging on pre-leukaemic cells. **a**, Experimental design to examine the effect of aging on pre-leukaemic cell expansion. Chimeric mice were generated using either young (6–10 weeks) or old (>52 weeks) recipient mice. Secondary transplants were performed with purified donor HSCs (CD45.2+). **b**, Absolute number of donor HSCs in the BM. Error bars represent the SEM (*n* = 9 mice per group). **c**, Donor-derived proportions in PB at the indicated time points (*n* = 9 mice per group). **d,e**, Bacterial 16S gene copies measured by qPCR in PB of young mice (6–12 weeks) 1 week post-DSS treatment (d) (*n* = 6 mice per group), and of young (6–10 weeks) or old mice (>52 weeks) (e) (Young, *n* = 10 mice per group; Old, *n* = 14 mice per group). **f**, Taxonomic composition of major bacterial phyla in mouse faecal, PB plasma, and BM homogenate samples of indicated WT mice (*n* = 3 biological replicates). Refer to Supplementary Table 2 for complete analysis. **g**, Summary of the combined average relative abundance of gram-negative (G-negative) and gram-positive (G-positive) bacterial taxa from the indicated samples (faeces, PB, and BM), related to Extended Data Fig. 3f (*n* = 9 biological replicates). **h**, Taxonomic composition of major bacterial phyla in human plasma samples of indicated patients. Refer to Supplementary Table 3 for complete analysis. **i**, Average relative abundance of gram-negative (G-negative) and gram-positive (G-positive) bacterial taxa related to Extended Data Fig. 3h (*n* = 8 biological replicates). *, *P* < 0.05, **, *P* < 0.01, ***, *P* < 0.001, ****, *P* < 0.0001. Error bars represent the SEM. *P* values were calculated for the indicated comparisons using a two-tailed unpaired Student’s t-test (b, d, e, g, and i) and a two-way ANOVA (c). Gram-negative, G-negative; Gram-positive, G-positive. Source data

Intestinal epithelial injury often results in disequilibrium in the bacterial ecosystem^25^. To investigate whether DSS-induced microbial dysbiosis drives *Dnmt3a*^*−/−*^ HSC expansion, we first performed faecal microbiota transplantation by exchanging intestinal microbiota from wild-type mice treated with water or DSS (Fig. 1f and Extended Data Fig. 2f). The microbial disequilibrium, characterized by an increased relative abundance of Gram-negative bacterial phyla, was preserved in the recipient mice after faecal transplantation (Extended Data Fig. 2g and Supplementary Table 1). Transplantation of microbiota from DSS-treated wild-type mice, but not from water-treated mice, resulted in the expansion of *Dnmt3a*^*−/−*^ HSCs (Fig. 1g). As ageing disrupts gut homeostasis^26^, we next examined the effects of age-associated bacterial disequilibrium on *Dnmt3a*-mutant HSCs. The intestinal permeability in aged mice was increased compared with in young mice (Extended Data Fig. 2h). Engraftment of *Dnmt3a*^*−/−*^ BM cells into old recipient mice that were conditioned with low-dose radiation resulted in an expansion of mutant HSCs and a significant increase in PB chimerism (Extended Data Fig. 3a–c), consistent with previous findings^27^. By contrast, *Dnmt3a*^*−/−*^ BM cells engrafted into young recipient mice conditioned with low-dose irradiation did not result in expansion of the mutant cells (Extended Data Fig. 3a–c), suggesting that age-related loss of intestinal epithelial integrity is a contributing factor to the *Dnmt3a*^*−/−*^ HSC expansion. To determine whether age-related bacterial disequilibrium contributes to pre-leukaemic cell expansion, we transplanted faecal material from young or old mice into mice engrafted with *Dnmt3a*^*−/−*^ BM cells (Fig. 1h). Microbiota from old mice resulted in the expansion of *Dnmt3a*^*−/−*^ HSCs, whereas microbiota from young mice did not (Fig. 1i). These findings demonstrate that intestinal barrier dysfunction and microbial alterations, whether from DSS or ageing, promote the selective expansion of *Dnmt3a*-mutant HSCs.

## Enrichment of ADP-heptose-producing microorganisms

**Fig. 2:**
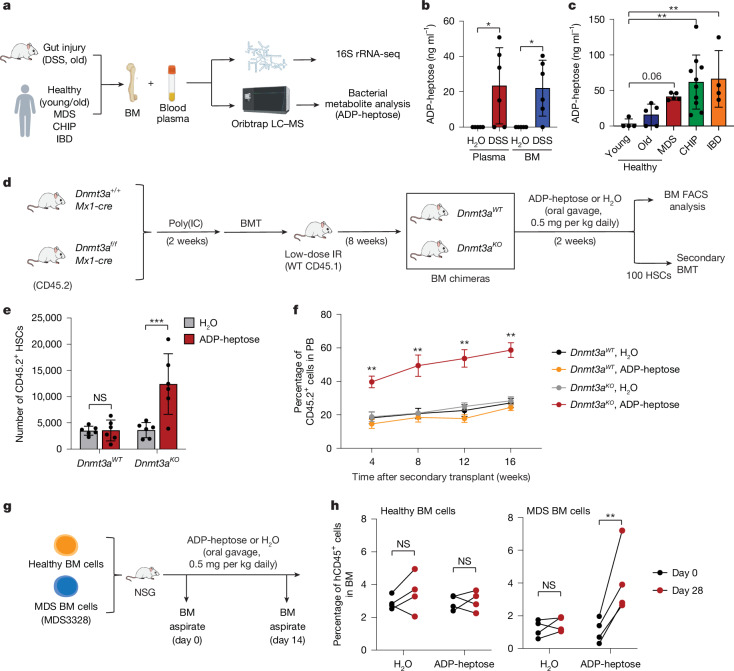
Circulating Gram-negative bacterial metabolite ADP-heptose contributes to pre-leukaemic HSC expansion. **a**, The experimental design to examine the microbiota through 16S rRNA-seq (Extended Data Fig. 3g,h) and metabolite analysis of the abundance of ADP-heptose (Fig. 2b,c) in mouse and human samples. **b**,**c**, ADP-heptose levels in mouse plasma and BM homogenates treated with H_2_O or DSS (*n* = 5 (H_2_O) and *n* = 6 (DSS) biological replicates) (**b**), and in human plasma samples (*n* = 4 (young), *n* = 5 (old), *n* = 5 (MDS), *n* = 10 (CHIP) and *n* = 4 (IBD) biological replicates) (**c**), as measured using MS. **d**, The experimental design to examine the effect of ADP-heptose treatment (adapted from Fig. 1a). Chimeric mice were treated with either H_2_O or ADP-heptose (0.5 mg per kg) for 2 weeks (*n* = 12 mice per group). Secondary transplants were performed with purified donor HSCs. **e**, The number of donor HSCs in the BM. *n* = 6 mice per group. **f**, Summary of donor-derived proportions in the PB at the indicated timepoints. *n* = 8 mice per group. Data are representative of three independent experiments. KO, knockout. **g**, Immunocompromised mice were xenografted with BM cells from one healthy individual and one patient with MDS (1 × 10^6^ donor cells per mouse) and then treated with either H_2_O or ADP-heptose (0.5 mg per kg) for 14 days. **h**, The percentage of human CD45^+^ cells in the BM of the indicated mice before and after treatment with ADP-heptose. *n* = 4 biological replicates per group from two independent experiments. Data are mean ± s.e.m. *P* values were calculated for the indicated comparisons using two-tailed unpaired Student’s *t*-tests (**b**,**c**,**e**) and two-way ANOVA (**f**,**h**). FACS-gating schemes are shown in Supplementary Fig. 2. The diagrams in **a** and **g** were created using BioRender. Source data

**Extended Data Fig. 4:**
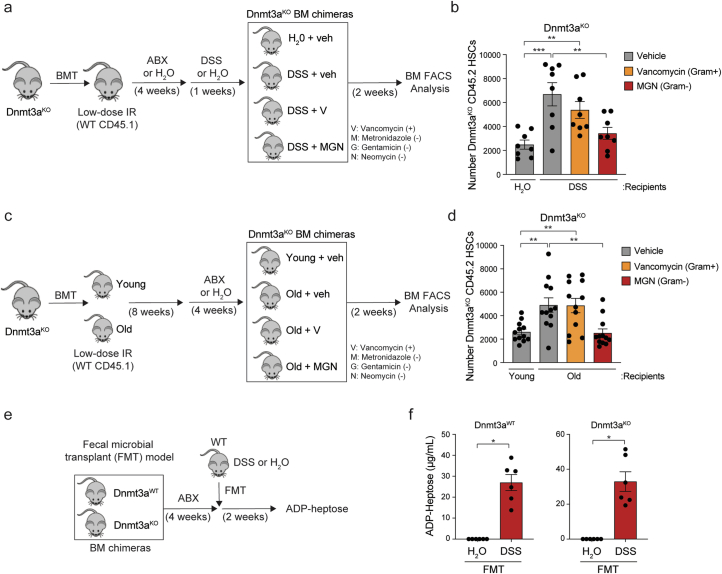
Gram-negative bacteria contribute to the expansion of pre-leukaemic HSCs. **a**, Experimental design to examine the contribution of bacteria (gram-positive vs gram-negative) following gut injury. BM cells from Dnmt3a^f/f^;Mx1-Cre mice (*n* = 4) treated with poly(IC) to induce recombination were transplanted into WT mice (*n* = 32) to generate chimeric mice. Chimeric mice established using low-dose irradiation were pre-treated with antibiotics targeting gram-negative bacteria (metronidazole, gentamicin, and neomycin) or antibiotics targeting gram-positive bacteria (vancomycin) for 4 weeks and then treated with DSS for 1 week. **b**, Number of donor HSCs (Lin-cKit+Sca1+ CD150+CD48-) in the BM 2 weeks after treatment (*n* = 8 mice per group). **c**, Experimental design to examine the contribution of bacteria (gram-positive vs gram-negative) in young and old mice. BM cells from Dnmt3a^f/f^;Mx1-Cre mice (*n* = 6) treated with poly(IC) to induce recombination were transplanted into WT mice (*n* = 48) to generate chimeric mice. Chimeric mice established using low-dose irradiation were pre-treated with antibiotics targeting gram-negative bacteria (metronidazole, gentamicin, and neomycin) or antibiotics targeting gram-positive bacteria (vancomycin) for 4 weeks. **d**, Number of donor HSCs (Lin-cKit+Sca1+ CD150+ CD48−) in the BM 2 weeks after treatment (n = 12 mice per group). **e**, Experimental design to examine the contribution of the microbiome using faecal microbial transplant (related to Fig. 1f,g). BM cells from Dnmt3a^f/f^;Mx1-Cre mice or Dnmt3a^+/+^;Mx1-Cre mice (*n* = 2 per group) treated with poly(IC) to induce recombination were transplanted into WT mice (*n* = 12 mice per group) to generate chimeric mice. Chimeric mice established using low-dose irradiation were pre-treated with broad-spectrum antibiotics (ABX) for 4 weeks and then colonized with faecal material from either H_2_O- or DSS-treated WT mice. **f**, ADP-heptose levels in plasma from Dnmt3a^WT^ or Dnmt3a^KO^ mice colonized by FMT from H_2_O- or DSS-treated WT mice (H_2_O: *n* = 6, DSS: *n* = 6 biological replicates). Error bars represent the SEM. *, *P* < 0.05, **; *P* < 0.01, ***; *P* < 0.001; ****, *P* < 0.0001. *P* values were calculated for the indicated comparisons using a two-tailed unpaired Student’s t-test (b, d, and f). Source data

Intestinal epithelial dysfunction, which is common in ageing, leads to dysbiosis and dissemination of microbial by-products^28^. To confirm this, we first measured the abundance of bacterial 16S rRNA gene in the PB (Fig. 2a). DSS-induced intestinal barrier dysfunction in mice increased 16S rRNA copies in the blood (Extended Data Fig. 3d). Increased bacterial 16S rRNA copies in the blood were also observed in old mice compared with in young mice (Extended Data Fig. 3e). Consistent with previous studies^28–30^, 16S rRNA gene sequencing showed that DSS-treated and aged mice had microbial alterations at the phylum level, including an approximately twofold increase in the relative abundance of Gram-negative bacteria in the PB plasma and BM (Extended Data Fig. 3f,g and Supplementary Table 2). Similarly, older humans exhibited an approximately threefold increase in the relative abundance of Gram-negative bacteria in the PB compared with young healthy individuals, consistent with previous studies^21,31^ (Extended Data Fig. 3h,i and Supplementary Table 3). As MDS is an ageing-related pre-leukaemic disease, we also sequenced 16S rRNA from the PB of age-matched individuals with MDS and found a comparable increased relative abundance of Gram-negative bacteria (Extended Data Fig. 3h,i and Supplementary Table 3), similar to previous studies linking Gram-negative bacteria enrichment to MDS outcomes^32^. The observed increases in the relative abundance of Gram-negative bacteria paralleled those in young individuals with IBD with severe intestinal epithelial injury (Extended Data Fig. 3h,i and Supplementary Table 3). Although we observed an increase in the relative abundance of Gram-negative bacteria in DSS-treated and aged mice and older humans, no specific disease- or age-associated bacterial species significantly expanded. To determine whether the increased abundance of Gram-negative bacteria contributes to *Dnmt3a*^*−/−*^ HSC expansion, we treated healthy recipient mice with antibiotics preferentially targeting Gram-negative (metronidazole, gentamicin and neomycin) or Gram-positive bacteria (vancomycin). Expansion of *Dnmt3a*^*−/−*^ HSCs in DSS-treated (Extended Data Fig. 4a,b) and aged recipient mice (Extended Data Fig. 4c,d) was suppressed with antibiotics targeting Gram-negative bacteria. By contrast, antibiotics targeting Gram-positive bacteria did not affect the expansion of *Dnmt3a*^*−/−*^ HSCs (Extended Data Fig. 4b,d). These findings suggest that age-associated intestinal barrier dysfunction and dysbiosis, characterized by Gram-negative bacterial enrichment, drive pre-leukaemic cell expansion.

## ADP-heptose expands pre-leukaemic cells

A hallmark of Gram-negative bacteria is their production of LPS. In contrast to LPS, which exhibits variable immunogenicity, ADP-heptose, a soluble LPS biosynthetic pathway intermediate, is highly immunogenic, released from live, lysed or internalized Gram-negative bacteria, and readily translocates across the plasma membrane to activate the cytosolic receptor ALPK1^3,4,19^. We confirmed ADP-heptose in the circulation by mass spectrometry (MS) after intestinal barrier dysfunction (Fig. 2a). Consistent with the increased dissemination of Gram-negative bacterial content during ageing, DSS-induced intestinal barrier dysfunction in mice resulted in circulating ADP-heptose in the BM and PB plasma (Fig. 2b). ADP-heptose was also detected in the circulation of wild-type and *Dnmt3a*^*−/−*^ recipient mice that were transplanted with microbiota from DSS-treated mice, but not in mice transplanted with microbiota from water-treated mice (Extended Data Fig. 4e,f). Older individuals and individuals with MDS also had detectable ADP-heptose in the plasma as compared to young individuals (Fig. 2c). Importantly, most young healthy individuals did not have detectable circulating ADP-heptose, indicating that dissemination of ADP-heptose is a consequence of age-dependent intestinal barrier dysfunction. Individuals with IBD of all ages also had circulating ADP-heptose in the PB plasma, consistent with severe epithelial injury (Fig. 2c and Supplementary Table 4).

**Extended Data Fig. 5:**
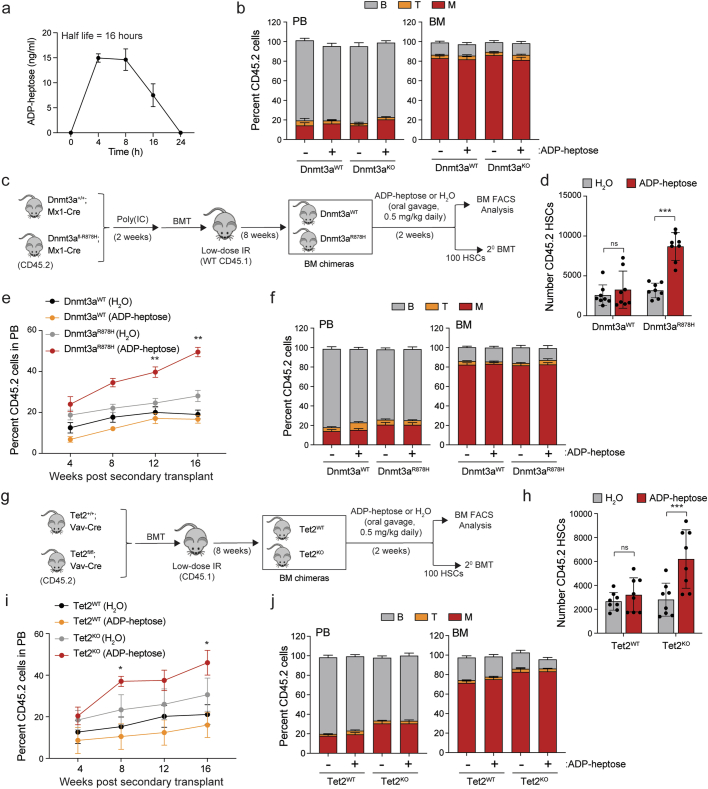
ADP-heptose induces pre-leukaemic cell expansion in vivo. **a**, Plasma concentration of ADP-heptose in mice at the indicated time points using mass spectrometry (*n* = independent replicates per time point). **b**, Myeloid (Gr1+ CD11b+) and lymphoid (CD19+ B220+ or CD3+) donor-derived proportions in PB and BM at 16 weeks (*n* = 8 mice per group). **c**, Experimental design to examine the effect of ADP-heptose. Chimeric mice were established by engrafting Dnmt3a^WT^ or Dnmt3a^R^^878H^ BM cells into WT recipient mice using low-dose irradiation and then treating with water (H_2_O) or ADP-heptose (0.5 mg/kg) for 2 weeks. Secondary transplants were performed with purified donor HSCs (CD45.2+). **d**, Number of donor HSCs (Lin-cKit+Sca1+ CD150+ CD48−) in the BM (*n* = 8 mice per group). **e**, Donor-derived proportions in PB at the indicated times (*n* = 8 mice per group). **f**, Myeloid and lymphoid donor-derived proportions in the PB and BM at 16 weeks (*n* = 8 mice per group). **g**, Chimeric mice were established by engrafting Tet2^WT^ or Tet2^−/−^ BM cells into WT recipient mice using low-dose irradiation and then treating with water (H_2_O) or ADP-heptose (0.5 mg/kg) for 2 weeks. Secondary transplants were performed with purified donor HSCs. **h**, Number of donor HSCs in the BM (*n* = 8 mice per group). **i**, Donor-derived proportions in PB at the indicated time points (*n* = 8 mice per group). **j**, Myeloid and lymphoid donor-derived proportions in the PB and BM at 16 weeks (*n* = 8 mice per group). Error bars represent the SEM. *, *P* < 0.05, **, *P* < 0.01, ***, *P* < 0.001. *P* values were calculated for the indicated comparisons using a two-tailed unpaired Student’s t-test (a, d, and h) and a two-way ANOVA (b, e, f, i, and j). B, B cells; T, T cells; M, myeloid cells. Source data

Loss of intestinal epithelial integrity, such as caused by ageing, resulted in the circulation of the Gram-negative bacterial metabolite ADP-heptose. To determine whether ADP-heptose drives *Dnmt3a*^*−/−*^ HSC expansion, we engrafted *Dnmt3a*^*−/−*^ or wild-type BM cells into low-dose-irradiated mice and then treated them with ADP-heptose (Fig. 2d). The selected concentration of ADP-heptose approximated plasma levels found in older individuals and was determined based on its half-life in healthy mice (Extended Data Fig. 5a). ADP-heptose administration resulted in *Dnmt3a*^*−/−*^ HSC expansion (Fig. 2e) and sustained self-renewal, evident by increased PB chimerism and multilineage differentiation in secondary recipient mice (Fig. 2f and Extended Data Fig. 5b). By contrast, ADP-heptose did not affect wild-type HSC numbers and engraftment in secondary recipient mice. We next determined whether ADP-heptose affects other CHIP-associated mutations, including loss-of-function *TET2* and *DNMT3A* mutations (DNMT3A(R882H) in humans or DNMT3A (R878H) in mice^33^). Like *Dnmt3a*^*−/−*^ HSCs, ADP-heptose expanded *Tet2*^*−/−*^ and *Dnmt3a*^*R878H/+*^ HSCs, and promoted self-renewal and multilineage differentiation (Extended Data Fig. 5c–j). To determine the effects of ADP-heptose on human pre-leukaemic cells, we examined primary human MDS BM-derived hematopoietic stem and progenitor cells (HSPCs) in vivo (Fig. 2g). MDS BM cells xenografted in immunocompromised mice expanded after 28 days of ADP-heptose administration, whereas ADP-heptose did not affect the engraftment of healthy BM cells (Fig. 2h). These findings demonstrate that circulating ADP-heptose drives expansion of both mouse and human pre-leukaemic cells.

## ADP-heptose forms TIFAsomes in pre-leukaemia

**Fig. 3:**
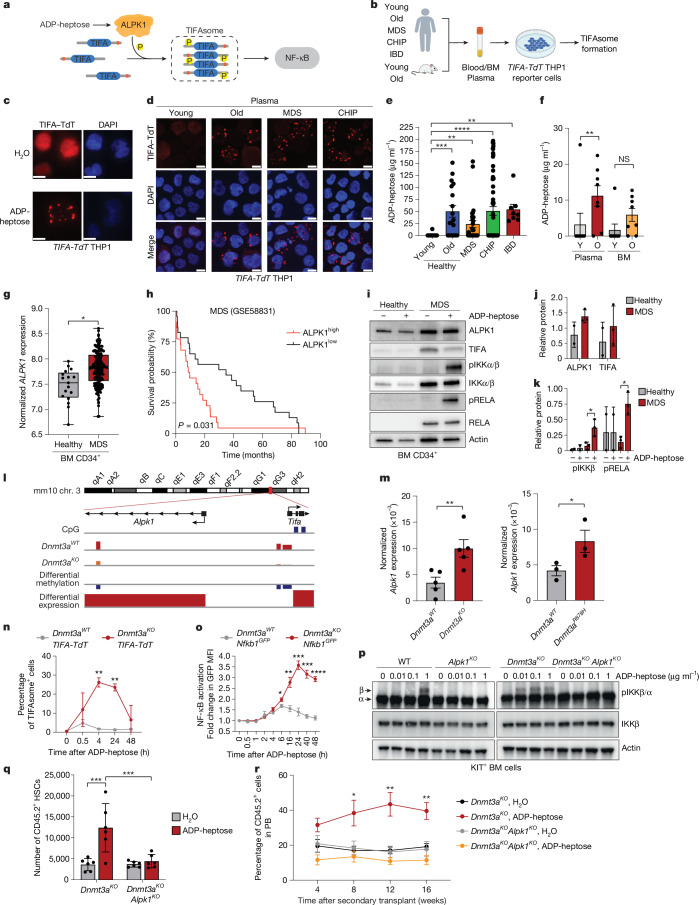
ADP-heptose induces TIFAsome activation and requires ALPK1 in pre-leukaemic cells. **a**, ADP-heptose-mediated activation of ALPK1 signalling. **b**, TIFAsome-formation assay in THP1 cells expressing *TIFA-TdT* to assess ADP-heptose in the plasma. **c**,**d**, *TIFA-TdT* THP1 cells were treated with ADP-heptose (1 µg ml^−1^) (**c**) or with human plasma (**d**). Scale bars, 10 µm. Data are representative of four biological replicates. **e**, ADP-heptose in the plasma of healthy young (<65 years, *n* = 11) and older (≥65 years, *n* = 18) individuals, and individuals with MDS (*n* = 29), CHIP (*n* = 59) or IBD (*n* = 8). **f**, ADP-heptose in matched BM and PB plasma of young (6–10 weeks, *n* = 8) or old (>52 weeks, *n* = 8) mice. **g**, *ALPK1* expression in healthy individuals (*n* = 17) or individuals with MDS (*n* = 159). The box plots show the median (centre line), the 25th and 75th percentiles (box limits) and the minimum and maximum values (whiskers). **h**, Survival stratified on *ALPK1* expression (highest and lowest 25%). *n* = 22 per group. **i**, Immunoblot analysis of cells stimulated with ADP-heptose (1 µg ml^−1^) for 30 min. **j**,**k**, Quantification from **i** (**j**) and Extended Data Fig. 7b (**k**). *n* = 3 biological replicates. **l**, Differential methylation of *Alpk1* and *Tifa* on mouse chromosome 3. **m**, *Alpk1* expression in HSCs. *n* = 5 (left) and *n* = 3 (right). **n**, TIFAsomes in *TIFA-TdT* KIT^+^ cells after ADP-heptose (1 µg ml^−1^) treatment. *n* = 3 biological replicates. **o**, The GFP mean fluorescence intensity (MFI) in HSCs after ADP-heptose (1 μg ml^−1^) treatment. *n* = 3 biological replicates. **p**, Immunoblot analysis of HSPCs after ADP-heptose (1 µg ml^−1^) treatment for 30 min. Representative blot from three biological replicates. **q**, Donor HSCs in the BM. *n* = 6 biological replicates. **r**, Donor-derived chimerism in the PB. *n* = 8 mice. Data are mean ± s.e.m. *P* values were calculated using two-tailed unpaired Student’s *t*-tests (**e**–**g**,**m**,**q**) and two-way ANOVA (**n**,**o**). Uncropped blots and FACS-gating schemes are shown in Supplementary Figs. 1 and 2, respectively. The diagram in **b** was created using BioRender. Source data

**Extended Data Fig. 6:**
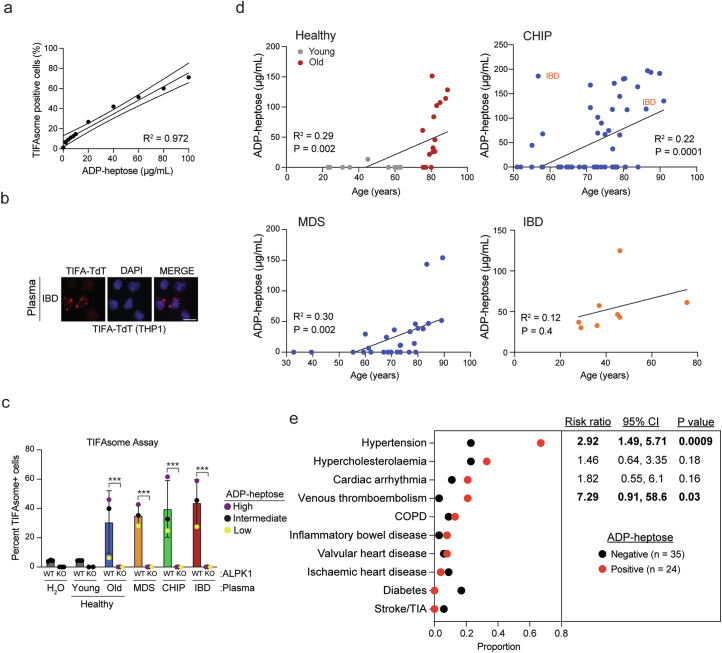
Circulating ADP-heptose in plasma is sufficient to induce TIFAsome formation in human pre-leukaemic cells. **a**, Standard curve generated by measuring TIFAsome formation by THP1-TIFA-tdTomato-GFP cells when stimulated with increasing concentrations of ADP-heptose from 0–100 µg/mL for 30 min, and evaluated on the ImageStream analyzer. **b**, Representative TIFAsome formation by THP1-tdTomato cells when stimulated with the plasma of IBD patients. Shown is a representative result of 3 independent replicates. Scale bar, 10 µM. **c**, TIFAsome formation assay using THP1-TIFA-tdTomato-GFP cells (WT and ALPK1-deficient) represented as percent positive cells following incubation with the indicated human plasma samples (*n* = 3 from independent biological replicates). **d**, Correlation of plasma ADP-heptose levels as extrapolated from the TIFAsome formation assay with the age of healthy young (< 65 years, *n* = 11), old (≥65 years, *n* = 18), MDS (*n* = 29), CHIP (*n* = 59), and IBD (*n* = 8) patients. **e**, Risk assessment of CHIP individuals with positive (*n* = 24) or negative (*n* = 35) ADP-heptose in circulation. Error bars represent the SEM. *, *P* < 0.05, **, *P* < 0.01, ***, *P* < 0.001. *P* values were calculated for the indicated comparisons using a two-tailed unpaired Student’s t-test (d and e) and a two-way ANOVA (c). FACS-gating schemes are shown in Supplementary Fig. 2. Source data

**Extended Data Fig. 7:**
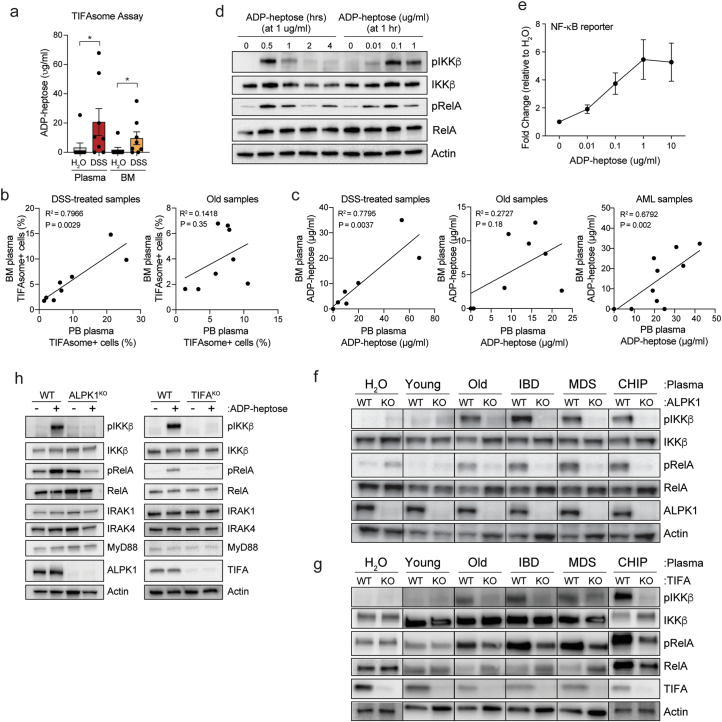
ADP-heptose-mediated TIFAsome signalling. **a**, Abundance of ADP-heptose in matched BM and PB plasma samples of H_2_O- or DSS-treated mice as measured by the TIFAsome formation assay (*n* = 8 mice per group). **b**, Correlation of TIFAsome-positive THP1 cells expressing TIFA-tdTomato in matched BM and PB plasma samples (*n* = 8 mice per group). **c**, Correlation of ADP-heptose levels (as calculated from the TIFAsome assay) in matched BM and PB plasma samples from DSS-treated mice (n = 8 mice per group), aged mice (*n* = 8 mice per group), and AML patients (*n* = 15 patients). **d**, Immunoblotting of THP1 cells treated with ADP-heptose at the indicated times and concentrations to determine NF-κB activation. **e**, THP1-NF-κB reporter cells were stimulated with indicated concentrations of ADP-heptose and NF-κB activity assessed after 24 h (*n* = 4 independent biological replicates). **f**, Immunoblotting of isogenic THP1-TIFA-tdTomato-ALPK1^WT^ and -ALPK1^KO^ cells treated with H_2_O or indicated human plasma samples for 30 min. **g**, Immunoblotting of isogenic THP1-TIFA-tdTomato-TIFA^WT^ and -TIFA^KO^ cells treated with H_2_O or indicated human plasma samples for 30 min. **h**, Immunoblotting of isogenic THP1-TIFA-tdTomato-ALPK1^WT^ and -ALPK1^KO^ or -TIFA^WT^ and -TIFA^KO^ cells untreated or treated with ADP-heptose (1 µg/ml) for 30 min. For gel source data, see Supplementary Fig. 1. Error bars represent the SEM. *, *P* < 0.05. *P* values were calculated for the indicated comparisons using a two-tailed unpaired Student’s t-test (a, b, and c) and a two-way ANOVA (e). Source data

A previous study^3^ showed that ADP-heptose binds to the cytosolic atypical kinase ALPK1, a conserved bacterial surveillance mechanism, activating TNF-receptor-associated factor (TRAF)-interacting protein with a forkhead-associated domain (TIFA), forming oligomeric TIFAsomes and inducing NF-κB activation^34^ (Fig. 3a). To assess whether circulating ADP-heptose in the plasma is sufficient to induce TIFAsomes in leukaemic cells, we generated human leukaemic cells expressing TIFA fused to TdTomato (*TIFA-TdT* THP1 cells), enabling visualization using fluorescence microscopy (Fig. 3b). While TIFA–TdT is cytoplasmically diffuse, ADP-heptose rapidly induced TIFAsomes as indicated by discrete puncta in the cytoplasm (Fig. 3c). Using integrated flow cytometry and fluorescence microscopy (Extended Data Fig. 6a), we confirmed that plasma from individuals with CHIP and MDS and older individuals induced TIFAsome formation in the *TIFA-TdT* THP1 cells (Fig. 3d,e and Supplementary Table 5), suggesting that circulating levels of ADP-heptose can readily activate ALPK1. TIFAsome formation was also induced by plasma from older individuals (Fig. 3e,f). Importantly, plasma from young healthy individuals was unable to induce TIFAsomes (Fig. 3d,e), confirming that there are insufficient circulating levels of ADP-heptose to activate ALPK1 in leukaemic cells. As a positive control, plasma from young individuals with IBD also induced TIFAsomes (Fig. 3e and Extended Data Fig. 6b). TIFAsome formation induced by the plasma was dependent on ALPK1 as ALPK1-deficient *TIFA-TdT* THP1 cells were unable to form TIFAsomes (Extended Data Fig. 6c). In all cases (besides individuals with IBD), TIFAsome formation correlated with age (Extended Data Fig. 6d and Supplementary Table 5), suggesting that circulating ADP-heptose occurs after ageing and reaches levels in individuals with CHIP or with MDS sufficient to activate ALPK1 in pre-leukaemic cells. ADP-heptose levels and TIFAsome formation was also associated with hypertension (risk ratio = 2.9; 95% confidence interval (CI) 1.5–5.7; *P* = 0.0009) and venous thromboembolism (risk ratio = 7.3; 95% CI 0.9–58.6; *P* = 0.03) in individuals with CHIP (Extended Data Fig. 6e and Supplementary Table 5). Moreover, TIFAsome formation was induced in *TIFA-TdT* THP1 cells by PB plasma from aged but not young mice (Fig. 3f) and in DSS-treated mice (Extended Data Fig. 7a). ADP-heptose levels and TIFAsome formation were comparable in the PB and BM plasma of aged and DSS-treated mice (*R*^2^ = 0.142–0.797) and in individuals with leukaemia (*R*^2^ = 0.6792), indicating systemic circulation of ADP-heptose (Fig. 3f and Extended Data Fig. 7b,c). These findings suggest that intestinal barrier dysfunction and enrichment of Gram-negative bacteria, as occurs in ageing, correlate with circulating ADP-heptose and TIFAsome formation in pre-leukaemic cells, potentially altering immune and inflammatory states in CHIP.

As TIFAsomes can initiate NF-κB activation^34^, we confirmed that ADP-heptose induces NF-κB signalling and transcriptional activation in human leukaemic cells (Extended Data Fig. 7d,e). To determine whether TIFAsomes induced by circulating ADP-heptose can initiate downstream pathway activation in leukaemic cells, we next evaluated NF-κB signalling after treatment with plasma derived from individuals classified as young, old and those with CHIP, as well as individuals diagnosed with MDS and IBD. Plasma from young healthy individuals did not induce NF-κB activation in leukaemic cells, as indicated by a lack of phosphorylated IKKβ and RELA (Extended Data Fig. 7f,g). However, plasma from older individuals, and individuals with CHIP, MDS and IBD induced robust NF-κB activation (Extended Data Fig. 7f,g). NF-κB activation in leukaemic cells required ADP-heptose-induced TIFAsomes as ALPK1- and TIFA-deficient THP1 cells did not activate NF-κB when incubated with patient plasma (Extended Data Fig. 7f,g) or directly with ADP-heptose (Extended Data Fig. 7h). These data demonstrate that age-associated circulating ADP-heptose is sufficient to induce TIFAsomes and NF-κB in pre-leukaemic cells.

## ADP-heptose requires ALPK1 in pre-leukaemia

**Extended Data Fig. 8:**
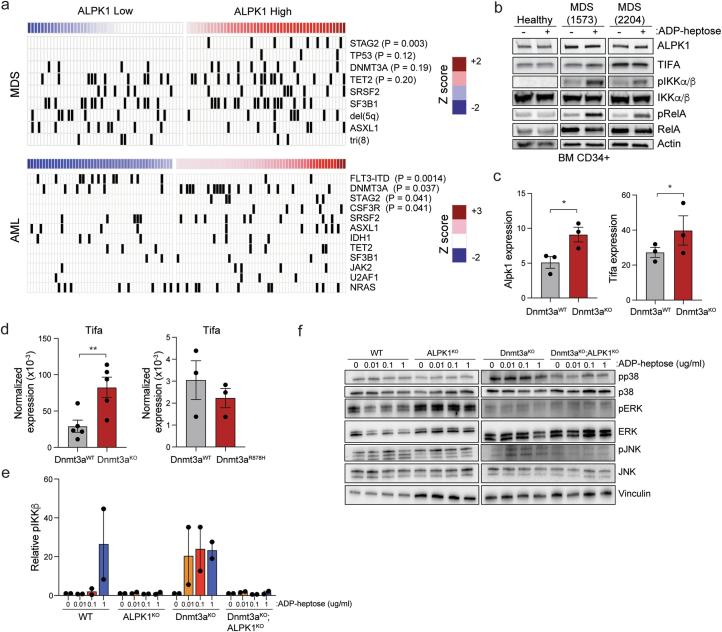
ALPK1 expression in human MDS/AML and Dnmt3a mutant HSPCs. **a**, Enrichment of MDS- and AML-associated mutations in patients based on high or low levels of ALPK1 (Z score). **b**, Immunoblotting of BM CD34+ cells isolated from healthy donor (*n* = 1) and MDS patients (*n* = 2) stimulated with ADP-heptose (1 µg/ml) for 30 min. **c**, ALPK1 and TIFA mRNA expression in Dnmt3a^WT^ and Dnmt3a^KO^ HSC from the publicly available dataset GSE98191 (*n* = 3 mice per group). **d**, Relative mRNA expression of Tifa in purified HSCs isolated from matched Dnmt3a^WT^ and Dnmt3a^KO^ (*n* = 5 mice per group) or Dnmt3a^WT^ and Dnmt3a^R878H^ mice (*n* = 3 mice per group). **e**, Quantification of immunoblotting from Fig. 3p (*n* = 2 independent experiments). **f**, Immunoblot analysis of HSPCs from WT, Alpk1K^O^, Dnmt3a^KO^, and Dnmt3a^KO^;Alpk1^KO^ mice stimulated with indicated concentrations of ADP-heptose for 30 min to examine MAPK signalling (*n* = 2 independent experiments). For gel source data, see Supplementary Fig. 1. Error bars represent the SEM. *, *P* < 0.05, **, *P* < 0.01, ***, *P* < 0.001. *P* values were calculated for the indicated comparisons using a two-tailed unpaired Student’s t-test (c, d). Source data

As ALPK1 is the only known ADP-heptose receptor in mammalian cells, we examined its expression in pre-leukaemic cells. BM-derived CD34^+^ HSPCs from individuals with MDS showed significantly higher *ALPK1* mRNA levels compared with the age-matched controls (Fig. 3g), correlating with a worse prognosis (Fig. 3h). Individuals with MDS (*P* = 0.18) and AML (*P* = 0.03) with high *ALPK1* expression had more *DNMT3A* mutations (Extended Data Fig. 8a). However, *ALPK1* expression was also increased in individuals without *DNMT3A* mutations, suggesting that activated TIFAsomes may involve other leukaemia driver mutations. Immunoblotting confirmed increased ALPK1 protein in MDS BM CD34^+^ HSPCs compared with cells from normal donors (Fig. 3i,j and Extended Data Fig. 8b). ADP-heptose induced TIFA-dependent NF-κB activation, as indicated by phosphorylated IKKβ and RELA, in MDS but not healthy CD34^+^ HSPCs (Fig. 3i,k and Extended Data Fig. 8b). These findings suggest mutant HSCs gain *ALPK1* expression, enabling ADP-heptose sensing. Aged and pre-leukaemic HSCs exhibit aberrant methylation, including *DNMT3A*-mutant pre-leukaemic cells^22^. Using publicly available data^23^, we found that *Dnmt3a*^*−/−*^ HSCs showed *ALPK1* promoter hypomethylation and increased *Alpk1* mRNA expression (Fig. 3l,m and Extended Data Fig. 8c). Similarly, *Dnmt3a*^*R878H/+*^ HSCs had increased *Alpk1* expression (Fig. 3m). Given *Tifa* promoter hypomethylation in *Dnmt3a*^*−/−*^ HSCs, *Tifa* mRNA was also increased compared with in wild-type HSCs (Extended Data Fig. 8d).

We observed that intestinal epithelial injury and dysbiosis mediate the expansion of *Dnmt3a*-mutant pre-leukaemic cells; we therefore next evaluated whether ADP-heptose drives *Dnmt3a*-mutant HSC expansion through ALPK1 activation. Treatment of *Dnmt3a*^*−/−*^ HSPCs expressing *TIFA-TdT* (*Dnmt3a*^*−/−*^*TIFA-TdT*) with ADP-heptose resulted in robust and sustained TIFAsomes as compared to wild-type *Dnmt3aTIFA-TdT* HSPCs (Fig. 3n). TIFAsome formation correlated with NF-κB activation in *Dnmt3a*^*−/−*^ HSCs expressing the enhanced green fluorescent protein (eGFP) reporter under transcriptional control of NF-κB *cis* motifs (*Dnmt3a*^*−/− *^*cis*-NF-κB^eGFP^) but to a lesser extent in wild-type *Dnmt3a* *cis*-NF-κB^eGFP^ HSCs (Fig. 3o). ADP-heptose activated NF-κB (pIKKβ) in *Dnmt3a*^*−/−*^ HSPCs at ≥0.01 µg ml^−1^, while wild-type HSPCs required higher concentrations of ADP-heptose (≥1 μg ml^−1^) (Fig. 3p and Extended Data Fig. 8e). NF-κB activation required ALPK1, as *Dnmt3a*^*−/−*^*Alpk1*^*−/−*^ HSPCs did not respond to ADP-heptose (Fig. 3p and Extended Data Fig. 8e). Simultaneous activation of NF-κB and MAPK, such as during chronic inflammation with IL-1β, promotes differentiation of HSCs at the expense of self-renewal, leading to depletion of the HSC population^35^. By contrast, ADP-heptose did not activate MAPK signalling in *Dnmt3a*^*−/−*^ HSPCs (Extended Data Fig. 8f), suggesting that its effects are specific to NF-κB signalling in pre-leukaemic cells.

**Extended Data Fig. 9:**
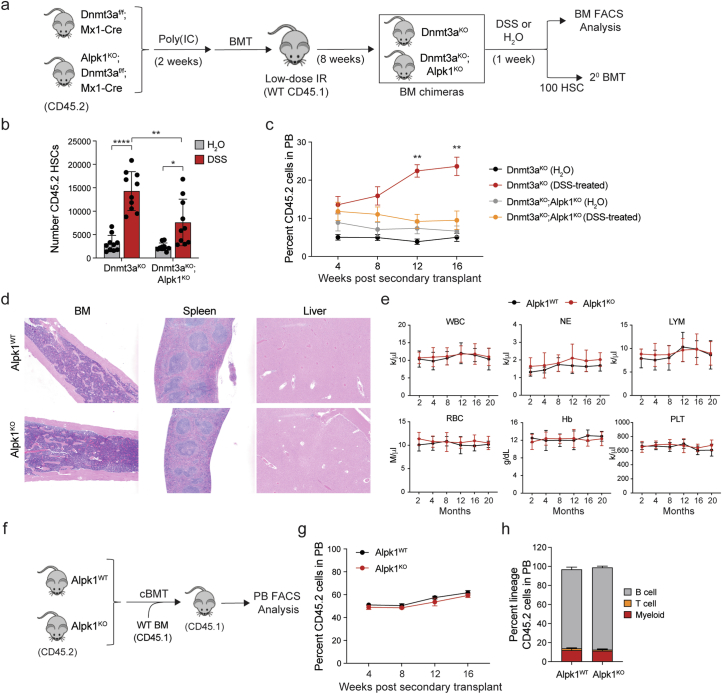
Loss of intestinal epithelial integrity contributes to expansion of pre-leukaemic cells via ALPK1. **a**, Experimental design to examine the effect of gut injury. BM cells from three Dnmt3a^f/f^;Mx1-Cre mice or three Dnmt3a^f/f^;Alpk1^KO^;Mx1-Cre mice treated with poly(IC) to induce recombination were transplanted into WT mice (*n* = 20 mice per group) to generate chimeric mice. Chimeric mice were treated with either water (H_2_O) or DSS (2.5%) for 1 week, and flow cytometry was performed on BM. Secondary transplants were performed with purified donor HSCs (CD45.2+). **b**, Number of donor HSCs (Lin-cKit+Sca1 + CD150 + CD48-) in the BM (*n* = 10 mice per group). **c**, Donor-derived proportions in PB at the indicated time points (*n* = 8 mice per group). **d**, H&E-stained BM, spleen, and liver isolated from Alpk1^WT^ (upper panel) and Alpk1^KO^ (bottom panel) mice. Images are representative of four replicates. **e**, Complete blood counts of Alpk1^WT^ and Alpk1^KO^ mice (*n* = 8 mice per group). WBC, white blood cells; NE, neutrophils; LYM, lymphocytes; RBC, red blood cells; Hb, haemoglobin; PLT, platelets. **f**, Outline of competitive BM transplantation experiment where BM cells from Alpk1^WT^ or Alpk1^KO^ mice (*n* = 1 mouse per group), and BM cells from one WT mouse (CD45.1) were transplanted competitively (1:1) into WT mice (CD45.1; total 1×10^6^ cells per mouse) (*n* = 6 mice per group). **g**, Donor-derived proportions in PB at the indicated time points (*n* = 6 mice per group). **h**, Donor-derived multilineages, B (CD19+ B220+), T (CD3+), and myeloid (Gr1+ CD11b+) cells in the PB (*n* = 6 mice per group). Error bars represent the SEM. *, *P* < 0.05, **, *P* < 0.01, ***, *P* < 0.001. *P* values were calculated for the indicated comparisons using a two-tailed unpaired Student’s t-test (b) and a two-way ANOVA (c, e, g, and h). Source data

Our model suggests that intestinal epithelial dysfunction and circulating ADP-heptose drive pre-leukaemic cell expansion through ALPK1-dependent signalling. To test this, *Dnmt3a*^*−/−*^ or *Dnmt3a*^*−/−*^*Alpk1*^*−/−*^ BM cells were engrafted into low-dose irradiated mice and then subsequently treated with DSS (Extended Data Fig. 9a). Expansion of *Dnmt3a*^*−/−*^*Alpk1*^*−/−*^ HSCs in the BM and their competitive advantage in secondary recipient mice after DSS treatment were significantly reduced as compared to *Dnmt3a*^*−/−*^ HSCs (Extended Data Fig. 9b,c). To confirm that the expansion of *Dnmt3a*^*−/−*^ HSCs is mediated by ADP-heptose, *Dnmt3a*^*−/−*^ or *Dnmt3a*^*−/−*^*Alpk1*^*−/−*^ BM cells were engrafted into low-dose-irradiated mice and then treated with ADP-heptose (as in Fig. 2d). Whereas ADP-heptose significantly expanded *Dnmt3a*^*−/−*^ HSCs and conferred a competitive advantage in secondary recipient mice, *Dnmt3a*^*−/−*^*Alpk1*^*−/−*^ HSCs did not expand or self-renew in recipient mice in response to ADP-heptose (Fig. 3q,r). *Alpk1*-deficient mice had no significant haematologic alterations, suggesting that ALPK1 is preferentially required in *Dnmt3a*-mutant HSCs (Extended Data Fig. 9d–h). These findings demonstrate that intestinal epithelial dysfunction and systemic ADP-heptose directly drive pre-leukaemic cell expansion through ALPK1.

## ADP-heptose alters signalling in pre-leukaemia

**Fig. 4:**
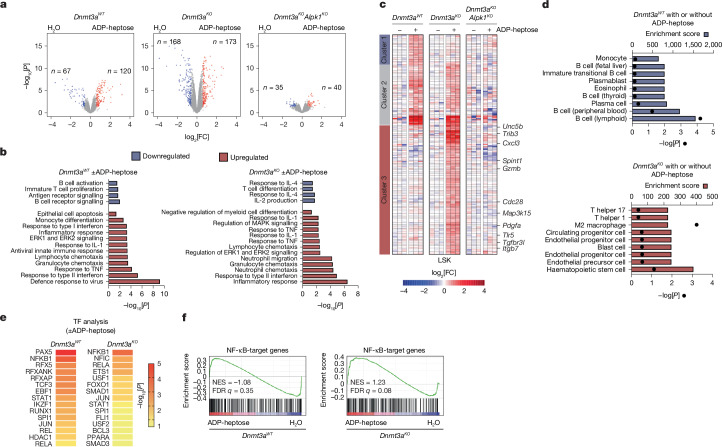
ADP-heptose induces transcriptional alterations in pre-leukaemic cells through ALPK1. **a**, All differentially expressed genes. Highlighted genes are more than twofold differentially expressed (adjusted *P* < 0.05) in ADP-heptose versus control (H_2_O-treated) HSPCs (Lin^−^KIT^+^SCA1^+^). *n* = 3 independent mice per group. **b**, Gene Ontology analysis of wild-type and knockout *Dnmt3a* cells treated with ADP-heptose versus H_2_O. **c**, Gene expression analysis of HSPCs isolated from wild-type or knockout *Dnmt3a* mice or double-knockout *Dnmt3a*^−/−^*Alpk1*^−/−^ mice treated with either H_2_O or ADP-heptose for 90 min in vitro from three independent replicates (1.5 fold; *P* < 0.05). **d**, Pathway enrichment of CellMarker datasets of upregulated genes in wild-type *Dnmt3a* cells treated with ADP-heptose versus H_2_O (left), and *Dnmt3a*-knockout cells treated with ADP-heptose versus H_2_O (right). The absolute enrichment score (ES) and the corresponding *P* value are shown for each pathway. The horizontal bars denote the enrichment score. The dots denote the *P* value. **e**, Enrichment of transcription factors was determined with the ENCODE and chromatin immunoprecipitation enrichment analysis (ChEA) libraries using genes that are overexpressed in ADP-heptose-stimulated wild-type *Dnmt3a* versus H_2_O-stimulated wild-type *Dnmt3a* cells (left), or ADP-heptose-stimulated *Dnmt3a*-knockout versus H_2_O-stimulated *Dnmt3a*-knockout cells (right). **f**, Gene set enrichment analysis of a curated list of NF-κB target genes (Supplementary Table 15) in wild-type and knockout *Dnmt3a* cells treated with ADP-heptose versus H_2_O. *P* values were calculated for the indicated comparisons using the Fisher’s exact tests (**b**,**d**).

**Extended Data Fig. 10:**
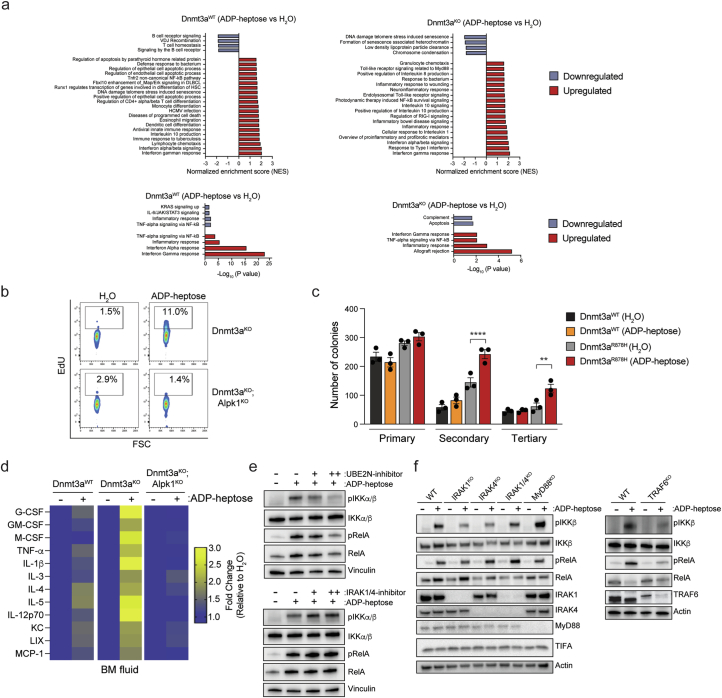
Effects of ADP-heptose on gene expression, inflammatory cytokines, and signalling pathways. **a**, Pathway enrichment of GSEA and Hallmark datasets in Dnmt3a^WT^ cells treated with ADP-heptose versus H_2_O (left panels) and Dnmt3a^KO^ cells treated with ADP-heptose versus H_2_O (right panels) (*n* = 3 mice per group). NES, normalized enrichment score. Absolute enrichment score (ES) and corresponding P value are shown for each pathway. **b**, Representative flow cytometric profile of EdU+ HSCs after ADP-heptose or H_2_O treatment. **c**, Serial colony replating of ADP-heptose- or H_2_O-treated BM HSPCs isolated from Dnmt3a^WT^ or Dnmt3a^R878H^ mice (*n* = 3 mice per group). In each plating, colonies were scored at day 14. **d**, Heatmap of differentially expressed cytokines and chemokines as measured in the BM fluid of Dnmt3a^WT^, Dnmt3a^KO^, and Dnmt3a^KO^;Alpk1^KO^ mice treated with ADP-heptose (0.5 mg/kg) in vivo (Data are representative of 4 independent biological replicates; for each replicate, BM was pooled from 2 different mice). **e**, Immunoblotting performed on THP1 cells untreated or treated with ADP-heptose (1 µg/ml) in combination with DMSO (-), UBE2N inhibitor at 5 µM (+), UBE2N inhibitor at 10 µM (+ +), IRAK1/4 inhibitor at 0.25 µM (+), and IRAK1/4 inhibitor at 0.5 µM (++) for 30 min. **f**, Immunoblot analysis of NF-κB modulators on wildtype (WT), IRAK1^KO^, IRAK4^KO^, IRAK1/4^dKO^, MyD88^KO^, and TRAF6^KO^ THP1 cells upon treatment with ADP-heptose (1 µg/ml) for 30 min. For gel source data, see Supplementary Fig. 1. Error bars represent the SEM. *, *P* < 0.05, **, *P* < 0.01, ***, *P* < 0.001. *P* values were calculated for the indicated comparisons using a two-way ANOVA (c). Source data

To investigate how ADP-heptose selectively expands pre-leukaemic cells, we performed global transcriptomic analysis on purified wild-type, *Dnmt3a*^*−/−*^ and *Dnmt3a*^*−/−*^*Alpk1*^*−/−*^ lineage^−^SCA1^+^KIT^+^ (LSK) HSPCs treated in vitro with ADP-heptose (Supplementary Tables 6–8). ADP-heptose induced significant gene expression changes in *Dnmt3a*^*−/−*^ HSPCs, with fewer differentially expressed genes in wild-type HSPCs (Fig. 4a). Most gene expression changes in *Dnmt3a*^*−/−*^ HSPCs were absent in *Dnmt3a*^*−/−*^*Alpk1*^*−/−*^ HSPCs (Fig. 4a), indicating that ADP-heptose is dependent on ALPK1. Gene Ontology analysis showed that ADP-heptose regulates pathways associated with innate immune signalling, inflammation and suppression of myeloid cell differentiation in *Dnmt3a*^*−/−*^ HSPCs (Fig. 4b, Extended Data Fig. 10a and Supplementary Tables 9–14). Many upregulated genes induced by ADP-heptose in *Dnmt3a*^*−/−*^ HSPCs are associated with immature haematopoietic cells and are known to increase cell proliferation and self-renewal programs, suggesting that ADP-heptose positively regulates leukaemic stem cell programs in pre-leukaemic HSCs (Fig. 4c,d). The differentially expressed genes induced by ADP-heptose in *Dnmt3a*^*−/−*^ HSPCs are enriched for NF-κB (NFKB1, RELA), HIF induction of nuclear factor I C (NFIC), STAT1 and ETS transcription factor DNA-binding motifs (Fig. 4e). By contrast, wild-type HSPCs stimulated with ADP-heptose expressed genes related to mature immune cells (Fig. 4d) and showed enrichment for PAX5, RFX5, TCF3 and STAT1 DNA motifs, with only a modest enrichment for NF-κB binding sites (Fig. 4e). Consistent with NF-κB motif enrichment and pathway activation (Fig. 3n,p), ADP-heptose-stimulated *Dnmt3a*^*−/−*^ HSPCs exhibited NF-κB target gene enrichment (normalized enrichment score (NES) = 1.23; false-discovery rate (FDR)-adjusted *q* = 0.08), while WT HSPCs did not (NES = −1.08; FDR-adjusted *q* = 0.35) (Fig. 4f and Supplementary Table 15). These findings suggest that *Dnmt3a*-mutant pre-leukaemic cells are primed to activate transcriptional programs associated with leukaemic states after ADP-heptose exposure.

## ADP-heptose directly expands pre-leukaemic HSCs

**Fig. 5:**
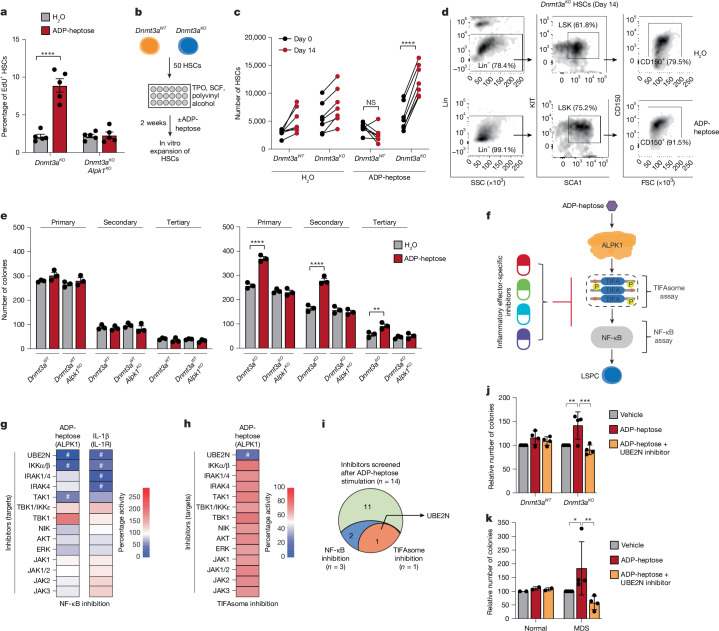
ADP-heptose induces pre-leukaemic cell proliferation and self-renewal through ALPK1-mediated NF-κB–UBE2N activation. **a**, The proportion of EdU-positive HSCs (Lin^−^KIT^+^SCA1^+^CD150^+^CD48^−^) within the BM of mice treated with EdU in vivo (after 2 weeks). *n* = 5 independent mice per group. **b**, The experimental design to assess the effect of ADP-heptose on HSC competition in vitro. **c**, The number of HSCs in H_2_O- or ADP-heptose-treated wells at day 0 and day 14 after treatment. *n* = 7 biological replicates per group from three independent experiments. **d**, Representative flow cytometry profile of HSCs (Lin^−^KIT^+^SCA1^+^CD150^+^) after 14 days. **e**, Serial colony replating of ADP-heptose- or H_2_O-treated BM HSPCs (Lin^−^SCA1^+^KIT^+^). Colonies were scored at day 14. *n* = 3 biological replicates per group. **f**, Overview of the screen using inhibitors targeting inflammatory-specific effectors in an NF-kB reporter cell line. **g**, THP1 NF-κB reporter cells were stimulated with either ADP-heptose or IL-1β in the presence of the indicated inhibitors for 24 h. *n* = 3 independent biological replicates. **h**, *TIFA-TdT* THP1 cells were treated with ADP-heptose (100 µg ml^−1^) in the presence of the indicated inhibitors for 24 h. *n* = 3 independent biological replicates. **i**, Prioritized hits in the NF-κB and TIFAsome assays. **j**, Colony-formation analysis of wild-type and knockout *Dnmt3a* HSPCs (Lin^−^KIT^+^SCA1^+^) after treatment with vehicle, ADP-heptose, or ADP-heptose and UBE2N inhibitor. *n* = 4 biological replicates per group. **k**, Colony-formation analysis of BM CD34^+^ cells from healthy individuals (normal; *n* = 2 technical replicates) and two individuals with MDS (*n* = 2 biological replicates) treated with vehicle, ADP-heptose, or ADP-heptose and UBE2N inhibitor. Data are mean ± s.e.m. The hash symbols (#) indicate greater than 50% inhibition. *P* values were calculated for the indicated comparisons using two-tailed unpaired Student’s *t*-tests (**a**) and two-way ANOVA (**c**,**e**,**j**,**k**). FACS-gating schemes are shown in Supplementary Fig. 2. The diagrams in **b** and **f** were created using BioRender. Source data

We observed that ADP-heptose drives pre-leukaemic cell expansion in vivo while activating both inflammatory and self-renewal gene expression programs. As inflammatory signalling can induce haematopoietic cell proliferation, we examined whether ADP-heptose stimulates *Dnmt3a*^*−/−*^ HSC proliferation in vivo. ADP-heptose significantly increased *Dnmt3a*^*−/−*^ HSC proliferation, which was abrogated in *Dnmt3a*^*−/−*^*Alpk1*^*−/−*^ HSCs (Fig. 5a and Extended Data Fig. 10b). At this timepoint, *Dnmt3a*^*−/−*^ HSCs expanded in the BM (Fig. 3q). Notably, *Dnm3a*^*−/−*^ HSCs exposed to ADP-heptose gained a long-term competitive advantage, as exhibited by increased PB chimerism and multilineage differentiation in secondary recipient mice (Fig. 3r). By contrast, the *Dnmt3a*^*−/−*^*Alpk1*^*−/−*^ or wild-type HSCs exposed to ADP-heptose did not expand or gain a competitive advantage (Figs. 3r and 5a). This suggests that ADP-heptose promotes *Dnmt3a*^*−/−*^ HSC proliferation without inducing precocious differentiation or stem cell exhaustion. To determine whether ADP-heptose acts directly on *Dnmt3a*^*−/−*^ HSCs, we conducted in vitro competition and progenitor self-renewal assays (Fig. 5b). Long-term in vitro HSC competition was assessed by co-culturing purified *Dnmt3a*^*+/+*^ (GFP^+^) and *Dnmt3a*^*−/−*^ (GFP^−^) HSCs in expansion medium containing polyvinyl alcohol and ADP-heptose for 14 days. While untreated *Dnmt3a*^*−/−*^ HSCs and wild-type HSCs maintained similar proportions (Fig. 5c), ADP-heptose treatment resulted in a competitive advantage of *Dnmt3a*^*−/−*^ HSCs relative to wild-type HSCs (Fig. 5c). Moreover, the proportion of immunophenotypically defined *Dnmt3a*^*−/−*^ HSCs increased after 14-day treatment with ADP-heptose as compared to control *Dnmt3a*^*−/−*^ HSCs, suggesting that ADP-heptose can expand and sustain *Dnmt3a*-mutant HSCs (Fig. 5d). The self-renewal potential of *Dnmt3a*^*−/−*^ HSCs was also promoted by ADP-heptose stimulation and dependent on ALPK1. ADP-heptose-treated *Dnmt3a*^*−/−*^ HSCs exhibited increased serial colony formation compared with vehicle-treated *Dnmt3a*^*−/−*^ HSCs (Fig. 5e), while *Dnmt3a*^*−/−*^*Alpk1*^*−/−*^ HSCs did not respond to ADP-heptose and formed colonies similar to wild-type HSCs treated with ADP-heptose (Fig. 5e). Similarly, ADP-heptose increased serial colony formation of *Dnmt3a*^*R878H/+*^ HSCs as compared to vehicle-treated mutant or wild-type HSCs (Extended Data Fig. 10c). These findings suggest that ADP-heptose confers a competitive advantage to pre-leukaemic cells through ALPK1-mediated transcriptional reprogramming.

## ADP-heptose activation of NF-κB uses UBE2N

Dysregulation of innate immune and inflammatory states contributes to pre-leukaemic conditions and leukaemia by creating an environment that suppresses normal haematopoiesis while promoting leukaemic stem and progenitor cell expansion^36^. Chronic inflammation in CHIP is also linked to cardiovascular disease^1^. The differentially expressed genes in ADP-heptose-treated *Dnmt3a*^*−/−*^ HSPCs were significantly enriched for NF-κB transcription factor binding (Fig. 4d,f). We therefore examined whether ADP-heptose induces inflammation and regulates pre-leukaemic cell expansion through NF-κB signalling. First, to determine whether ADP-heptose induces an inflammatory milieu, we treated *Dnmt3a*^*−/−*^ and wild-type mice with ADP-heptose and measured 32 cytokines, chemokines and growth factors in the BM fluid. ADP-heptose treatment of *Dnmt3a*^*−/−*^ mice resulted in elevated expression of numerous cytokines, including interleukin-1β (IL-1β), granulocyte colony-stimulating factor (G-CSF) and tumour necrosis factor (TNF) (Extended Data Fig. 10d), which are NF-κB target genes and are directly implicated in human disease and suppression of normal HSC function^35^. These findings suggest that ADP-heptose promotes pre-leukaemic cell expansion and systemic inflammation linked with cardiovascular disease.

NF-κB activation depends on upstream effectors that influence cancer cell proliferation and survival^37^. Distinct signalling inputs leading to NF-κB activation can impact the duration and amplitude of the signal, which determines whether NF-κB exerts tumour-promoting or suppressive effects^38^. To dissect TIFAsome-dependent NF-κB activation through ALPK1 in pre-leukaemic cells, we conducted an inhibitor screen targeting known NF-κB effectors (Fig. 5f). As expected, NF-κB activation through IL-1R requires TRAF6-dependent UBE2N and kinases IRAK1, IRAK4, TAK1 and IKKα/β (Fig. 5g). By contrast, ADP-heptose-induced NF-κB activation requires UBE2N and IKKα/β, but not IRAK1, IRAK4 and TAK1 (Fig. 5g and Extended Data Fig. 10e). Gene deletion studies confirmed that ADP-heptose-mediated activation of ALPK1 uses distinct effectors to initiate NF-κB activation compared with other inflammatory signals in pre-leukaemic cells (Extended Data Fig. 10f). In parallel, we screened the inhibitors for TIFAsome suppression (Fig. 5f). Only the UBE2N inhibitor prevented ADP-heptose-induced TIFAsomes (Fig. 5h), suggesting that UBE2N is essential for both ALPK1-dependent TIFAsome formation and NF-κB activation in leukaemic cells (Fig. 5i). To confirm the role of UBE2N in the expansion of leukaemic cells through ALPK1, we tested UBE2N inhibitors on mutant HSCs. Inhibition of UBE2N suppressed ADP-heptose-mediated colony formation of *Dnmt3a*^*−/−*^ HSPCs and MDS HSPCs in vitro (Fig. 5j,k). These findings demonstrate that ADP-heptose initiates TIFAsome formation and UBE2N-dependent activation of NF-κB, essential for expansion of pre-leukaemic cells.

## Discussion

Our results identified ADP-heptose as an age-associated microbial metabolite that promotes pre-leukaemic cell expansion. Ageing correlates with intestinal barrier dysfunction, enrichment of ADP-heptose-producing Gram-negative bacteria and circulating ADP-heptose, which enhances pre-leukaemic cell self-renewal and a competitive advantage over non-mutant haematopoietic cells. Individuals with pre-leukaemic conditions, such as CHIP or clonal cytopenias of unknown significance, are at increased risk of haematologic malignancies and early-onset cardiovascular conditions^6,39^. Although the absolute risk of leukaemic transformation for CHIP is low, the size of the mutant haematopoietic cell pool is a strong predictor of all-cause mortality. Despite advances in the understanding of the genetic and environmental factors contributing to CHIP, key drivers of pre-leukaemic cell expansion remain unclear. We show that systemic ADP-heptose, uniquely associated with ageing, promotes the expansion of pre-leukaemic cells, inflammation and cardiovascular disease risk. Notably, the aged microenvironment and the presence of ADP-heptose, rather than the age of the mutant cells, have a primary role in driving pre-leukaemic cell expansion.

We demonstrate that intestinal epithelial dysfunction and the enrichment of a broad ADP-heptose-producing Gram-negative bacterial consortium—rather than specific microbial taxa—occur during ageing and coincide with the translocation of ADP-heptose into circulation. In ageing, dysbiosis-like changes in microbiome composition are observed at the phylum and genus levels, characterized by an increase in Gram-negative bacteria, such as *Bacteroides*, and a depletion of Gram-positive bacteria, such as *Firmicutes*^40^. These changes also correlate with elevated inflammatory markers^31^. Moreover, impaired barrier function can increase gut oxygenation, favouring facultative anaerobes such as Enterobacteriaceae^41,42^, which may further exacerbate barrier dysfunction and contribute to pre-leukaemic cell expansion. Here we find that the expansion of pre-leukaemic cells is not mediated by specific bacterial taxa but, rather, by the overall relative increase in ADP-heptose-producing Gram-negative bacteria. This increase in ADP-heptose-producing Gram-negative bacteria can occur by expansion of a specific Gram-negative bacteria or by the relative increase in multiple Gram-negative bacterial taxa. Importantly, a combination of factors—loss of intestinal epithelial integrity, age-associated increase in Gram-negative bacteria (specific taxa or a broad relative increase) and circulating ADP-heptose—drives pre-leukaemic cell expansion. Furthermore, the pre-leukaemic cell expansion phenotype does not appear to be solely attributed to ‘unhealthy’ Gram-negative bacteria. Future studies should identify Gram-negative bacterial species that preferentially produce ADP-heptose and promote pre-leukaemic cell expansion. ADP-heptose is highly immunogenic, released from live or lysed Gram-negative bacteria, and freely translocates across the plasma membrane^3–5^. ADP-heptose is mostly absent in the circulation of young individuals but detectable in older individuals with CHIP and MDS. Furthermore, ADP-heptose in individuals with CHIP correlates with hypertension and venous thromboembolism risk. CHIP mouse models and human xenograft studies confirm that ADP-heptose provides *DNMT3A*-mutant pre-leukaemic cells with a competitive advantage, making ADP-heptose a dichotomic age-associated factor that drives pre-leukaemic cell expansion. Similar effects were observed in *TET2*-mutant cells, suggesting that ADP-heptose has a broader role in clonal haematopoiesis.

In a series of seminal studies, d-gylcero-d-manno-heptose-1,7,bisphosphate (HBP), a Gram-negative bacterial metabolite, was reported to induce TIFAsomes and immune responses^43–45^. ADP-heptose, derived from HBP, directly binds to ALPK1. Activated ALPK1 then phosphorylates TIFA at Thr9, leading to the formation of TIFAsomes^46^. Although ADP-heptose is implicated in Gram-negative bacterial-associated infections^5,47,48^, it remains to be determined whether specific species of Gram-negative bacteria produce higher levels of ADP-heptose than others, potentially due to variations in the regulation of ADP-heptose synthesis or differences in the gut milieu. In contrast to normal haematopoietic cells, ALPK1 and TIFA are highly expressed in pre-leukaemic cells. Loss-of-function studies in CHIP mouse models confirm that ALPK1 is essential for pre-leukaemic cells to detect circulating ADP-heptose and drive expansion.

As previous studies focused on ADP-heptose-induced ALPK1 activation in immune cells, we performed transcriptional analyses and inhibitor screens to define its role in pre-leukaemic expansion. ADP-heptose induces transcriptional programs that enhance self-renewal and proliferation of pre-leukaemic cells while preventing HSC exhaustion. In contrast to other inflammatory factors, ADP-heptose preserves pre-leukaemic HSCs in a proliferative state by avoiding stress-induced attrition. While canonical NF-κB signalling is often linked to stem cell exhaustion^49^, ADP-heptose-exposed pre-leukaemic cells continue proliferating. We hypothesize that the unique ability of ADP-heptose to preserve pre-leukaemic cells is due to its selective NF-κB activation without concurrent MAPK signalling. Moreover, ADP-heptose activates NF-κB through UBE2N, bypassing canonical inflammatory pathways. Disrupting this unique NF-κB signalling state prevents the ADP-heptose-induced pre-leukaemic cell expansion. *ALPK1* mutations have been linked to inflammatory disorders and cancer^50^, while our study implicates ALPK1 activation in ageing and pre-leukaemic conditions. We therefore propose the ADP-heptose–ALPK1 axis as a potential therapeutic target to prevent CHIP progression to overt leukaemia and immune-related conditions. These findings may have broader implications beyond haematology, potentially influencing our understanding of age-related and gut-associated diseases, including cancer and inflammatory disorders.

## Methods

### Human samples

Human CD34^+^ cells and cells from individuals with MDS were maintained in StemSpan Serum-Free Expansion Media (09650, StemCell Technologies) supplemented with 10 ng ml^−1^ of recombinant human stem cell factor (SCF) (300-07-50UG, PeproTech), recombinant human thrombopoietin (TPO) (300-18-50UG, PeproTech), recombinant human FLT3 ligand (FLT3L) (300-19-50UG, PeproTech), recombinant human IL-3 (200-03-50UG, PeproTech) and recombinant human IL-6 (200-06-50UG, PeproTech), as previously described^51^. Human CD34^+^ cells from healthy individuals were obtained from the Yale Cooperative Center of Excellence in Hematology (YCCEH). BM mononuclear cells from individuals with MDS (MDS3328) were obtained with written informed consent and approval of the institutional review board of the University of Cincinnati and Ohio State University and under the IRB-approved study ID 2008-0021. These samples had been obtained within the framework of routine diagnostic BM aspirations after written informed consent in accordance with the Declaration of Helsinki.

### Human plasma samples

Human plasma samples were obtained from multiple sources. Plasma from healthy individuals (young (<65 years), *n* = 5; old (≥65 years), *n* = 10) and individuals diagnosed with IBD (*n* = 8) or MDS (*n* = 9) were obtained from BioIVT. Plasma from individuals with MDS (*n* = 20) and AML (n = 15) was obtained from Ohio State University. Plasma from healthy individuals (young (<65 years), *n* = 6; old (≥65 years), *n* = 7) and individuals with IBD (*n* = 3) and CHIP (*n* = 29) were obtained from individuals undergoing elective total hip replacement surgery under the Mechanisms of Age-Related Clonal Haematopoiesis (MARCH) Study (NHS REC: 17/YH/0382) at the Oxford University Hospital, UK. Plasma from individuals with CHIP (*n* = 30) was obtained from the University of Cincinnati. All of the participants gave written informed consent in accordance with the Declaration of Helsinki. Detailed information is provided in Supplementary Tables 4 and 5.

### Cell lines

THP1 cells were purchased from American Type Culture Collection (ATCC). THP1 cells were cultured in RPMI-1640 medium (SH30027.01, HyClone) supplemented with 10% fetal bovine serum (FBS, S11550, Atlanta Biologicals) and 1% penicillin–streptomycin (SV30010, HyClone). HEK293T cells were cultured in Dulbecco’s modified Eagle medium (DMEM, 10-016-CV, Corning Cell Grow) supplemented with 10% FBS and 1% penicillin–streptomycin. THP1-NF-κB-Blue cells (thp-nfkb, Invivogen) were grown in complete THP1 medium with 100 µg ml^−1^ normocin and 10 µg ml^−1^ blasticidin added. As mentioned previously^52^, all cells were cultured at 37 °C and 5% CO_2_. Analysis of short tandem repeat loci (STR Profiling, ATCC, 135-XV-10) was performed on all cell lines when received and after experimentation was complete. Authentication reports are provided separately. All cell lines were routinely tested and were confirmed to be negative for mycoplasma.

### Inhibitors and reagents

Poly(I:C) (4287) was purchased from Tocris Bioscience. IL-1β (200-01B) was purchased from Peprotech. As previously published^53^, UC-764865 was initially obtained from the University of Cincinnati–Drug Discovery Center’s compound library and then synthesized and purchased from Wuxi AppTec. ADP-heptose (tlrl-adph-l), MRT67307 (inh-mrt) and Ultrapure-LPS (TLRL-PEKLPS) were purchased from Invivogen. GSK8612 (S8872) and ruxolitinib (S1378) were purchased from Selleckchem. *N*-Des (aminocarbonyl) AZ-TAK1 (ab143773) was purchased from Abcam. PF-06650833 (PZ0327-5MG) was purchased from Sigma-Aldrich. CA-4948 was purchased from ChemExpress. NIK SIM1 (HY-112433), AZD-1480 (HY-10193), itacitinib (HY-16997), tofacitinib (HY-40354), AKT inhibitor VIII (HY-10355) and trametinib (GSK1120212) were purchased from MedChem Express.

### Mice

*Dnmt3a*^*f/f*^ and *Mx1-cre*^*+*^ (obtained from H. L. Grimes laboratory)^54^, *Dnmt3a*^*fl-R878H*^ (B6(Cg)*-Dnmt3a*^*tm1Trow*^*/*J, 032289, Jackson Laboratory), *Tet2*^*fl/fl*^ (B6;129S*-Tet2*^*tm1.1Iaai*^*/*J, 017573, Jackson Laboratory), *Vav-cre*^*+*^ (B6.Cg*-Commd10*^*Tg(Vav1-icre)A2Kio*^*/*J, 008610, Jackson Laboratory), *Alpk1*^*−/−*^ (11 bp deletion in exon 3, C57BL/6N-*Alpk1*^*em1Fsha*^*/*J, 032561, Jackson Laboratory), *Tifa*^*−/−*^ (gift from J.-I. Inoue), and *UBC-GFP* (C57BL/6-Tg(UBC-GFP)30Scha/J, 004353, Jackson Laboratory) mice were maintained on a CD45.2^+^ C57BL/6 background. *cis-*NF-κB^eGFP ^reporter mice were provided by C. Jobin^55^. Throughout the study, CD45.1^+^ B6.SJL*-Ptprc*^*a*^/BoyJ mice were used as recipients for BM transplantation experiments. Littermate controls were used for all experiments. To generate *Dnmt3a*^*f/f*^*Mx1-cre*^*+*^ mice, *Dnmt3a*^*f/f*^ and *Mx1-cre*^*+*^ mice were crossed (referred to as *Dnmt3a*^*−/−*^ or *Dnmt3a*^*KO*^). To generate *Dnmt3a*^*−/−*^*Alpk1*^*−/−*^ mice, *Dnmt3a*^*−/−*^ and *Alpk1*^*−/−*^ mice were crossed. To generate *Dnmt3a*^*fl-R878H/+*^*Mx1-cre*^*+*^ mice, *Dnmt3a*^*fl-R878H/+*^ and *Mx1-cre*^*+*^ mice were crossed (referred to as *Dnmt3a*^*R878H/+*^). To generate *Tet2*^*f/f*^*Vav-cre*^*+*^ mice, *Tet2*^*f/f*^ and *Vav-cre*^*+*^ mice were crossed (referred to as *Tet2*^*−/−*^ or *Tet2* knockout). To generate *Dnmt3a*^*−/−*^* cis*-NF-κB^eGFP^ reporter mice, *Dnmt3a*^*−/−*^*Mx-cre*^*+*^ and *cis-*NF-κB^eGFP^ mice were crossed. All the mice carrying the *Mx1-cre* allele were given five doses of poly(I:C) every other day at 8–12 weeks of age. Animals of the same age and gender were randomly assigned to experimental groups. Investigators were not blinded.

### Husbandry and animal care

All mice were housed in the Association for Assessment and Accreditation of Laboratory Animal Care (AAALAC)-accredited animal facility at Cincinnati Children’s Hospital Medical Center, maintained under specific pathogen-free conditions and monitored under tightly controlled settings. They were housed on IVC racks (Allentown Jag 75 Micro-VENT Environmental Systems IVC racks) and kept in individually ventilated polysulfone shoebox cages (Alternative Design), with up to four mice per cage. The cages were supplied with corncob bedding (Bed-o’Cobs 1/4, The Andersons), ad libitum feed (LabDiet, 5010) and enrichment (Twist-n’Rich, The Andersons). All cage components were autoclaved before housing the mice, and cages were changed weekly. Mice had access to ad libitum water through a reverse osmosis autowater system. The mouse room was maintained on an automatic 12 h–12 h light–dark cycle at an ambient temperature of 23 °C and 30–70% humidity, and 5% Clidox-S was used as a disinfectant. Mice were bred, housed and monitored daily by laboratory staff and veterinary personnel to ensure good health, activity and the presence of appropriate food, water and cage conditions. Quarterly testing of pathogens was conducted in sentinel animals housed in the same room. Excluded agents included: *Mycoplasma pulmonis*, CAR bacillus, Ectromelia, rotavirus (EDIM), Hantaan virus, K virus, lymphocytic choriomeningitis virus, mouse adenoviruses (MAV1, MAV2), mouse cytomegalovirus, mouse hepatitis virus, mouse parvovirus, mouse thymic virus, minute virus of mice, polyoma virus, pneumonia virus of mice, reoviruses (REO3), Sendai virus, Theilers murine encephalomyelitis virus (TMEV), *Encephalitozoon cuniculi*, *Aspiculuris tetraptera*, Fur mites (Myocoptes, Radfordia/Myobia) and Pinworms (*Aspiculuris tetraptera*, *Syphacia muris*, *Syphacia obvelata*). All laboratory staff wore personal protective clothing, and all animal procedures were performed in accordance with the protocol approved by the Institutional Animal Care and Use Committee at Cincinnati Children’s Hospital (IACUC) (IACUC2019-0072). Procedures such as blood collection, faecal sample collection and oral gavage were conducted in a biosafety cabinet (NuAire) within the same experimental housing room (6445), eliminating the need to transport mouse cages through the halls during experiments. Throughout the study, care was taken to collect all biological specimens from the mice by a single scientist (K.H.) and processed by another scientist (P.A.) using the same laboratory equipment and reagent kits from the same commercial vendor.

### DSS and antibiotics treatment

Mice were treated with 2.5% DSS (w/v) (molecular mass: 36,000–50,000 Da, 216011090, MP Biomedicals) in autoclaved drinking water to induce gut injury-associated colitis, as described previously^56^. Control mice were time and anatomical location matched and received water only. Mice were monitored daily for weight loss, stool consistency and the presence of frank blood in the stool. Daily assessment of mortality/morbidity was performed, and mice were euthanized if they were in obvious distress (defined as immobility, weight loss >20% or severe bloody diarrhoea), and were therefore not included in the study. Study animals were allowed to recover on regular water for an additional 1–8 weeks. Blood was collected through the submandibular vein and faecal pellets, distal colons and BM were collected for histological analysis and flow cytometry. In parallel experiments, mice were pretreated with a broad-spectrum antibiotics cocktail to deplete endogenous host microbiota as previously described^57,58^. In brief, in the first week (Monday–Friday), mice received a daily oral gavage with 100 μl of antibiotics cocktail containing kanamycin (4 mg ml^−1^, Sigma-Aldrich, 60615), gentamicin (0.35 mg ml^−1^, Sigma-Aldrich, G1914), colistin (0.5 mg ml^−1^, Sigma-Aldrich, C4461), metronidazole (2.15 mg ml^−1^, Sigma-Aldrich, M3761) and vancomycin (0.45 mg ml^−1^, Sigma-Aldrich, V2002). For the next 3 weeks, antibiotics were administered in non-acidified autoclaved water at 0.2 mg ml^−1^ except for vancomycin, which was maintained at 0.5 mg ml^−1^. In few experiments wherever noted, in the first week (Monday–Friday), mice received a daily oral gavage of 100 μl of antibiotics containing either vancomycin (1 mg ml^−1^) to deplete Gram-positive bacteria or cocktail of metronidazole (2.15 mg ml^−1^), gentamicin (1 mg ml^−1^) and neomycin (1 mg ml^−1^) (MGN) to deplete Gram-negative bacteria. For the next 3 weeks, the antibiotics were administered in non-acidified autoclaved water maintained at 1 mg ml^−1^. Antibiotics water was prepared fresh and replaced weekly to supply fresh antibiotics.

### BM transplantation

To model pre-leukaemic clonal haematopoiesis, we generated chimeric mice as follows. In brief, a mixture of 1 × 10^6^ whole BM cells (WBM) was obtained from poly(I:C)-treated wild-type (*Dnmt3a*^*+/+*^*Mx1-cre*^*+*^, called *Dnmt3a* wild-type) or mutant mice (*Dnmt3a*^*f/f*^*Mx1-cre*^*+*^, called *Dnmt3a*^*KO*^) or double-mutant mice (*Dnmt3a*^*f/f*^*Mx1-cre*^*+*^*Alpk1*^*KO*^, called *Dnmt3a*^*KO*^*Alpk1*^*KO*^) (CD45.2^+^), and transplanted into low-dose (2.5 Gy) irradiated recipient mice (CD45.1^+^; 6–10 weeks of age). Then, 8 weeks after transplant, chimeric mice were treated with either water or DSS (2.5%) for 1 week, and allowed to recover for 1 more week on water after which flow cytometry was performed on the BM. In a separate experiment, chimeric mice were pretreated with broad-spectrum antibiotics for 4 weeks, and then subjected to DSS for 1 week after which flow cytometry performed on BM. In a separate set of experiments, chimeric mice were treated with either water or ADP-heptose (0.5 mg per kg) through oral gavage for 2 weeks, and flow cytometry analysis of the BM was performed after 2 more weeks. In all of the experiments, secondary transplantation was performed by purifying donor HSCs (CD45.2^+^Lin^−^KIT^+^SCA1^+^CD150^+^CD48^−^) and transplanting 100 HSCs with 200,000 helper WBM cells (CD45.1^+^) into lethally irradiated (8 Gy) recipient mice (CD45.1^+^), and donor chimerism in PB examined by flow cytometry.

### Quantification of bacterial DNA using qPCR

We used the previously described protocol to examine bacterial translocation into blood^59,60^. Whole blood was collected by cheek bleeding in sterile BD Microtainer Capillary Blood Collector and Microgard Closure tubes (13-680-62, Thermo Fisher Scientific) on ice from each mouse using Goldenrod Animal Lancets 4 mm (NC9922361, Braintree Scientific), and genomic DNA was extracted using the DNeasy Blood & Tissue Kit (69504, Qiagen). Quantitative PCR (qPCR) was performed using the Femto Bacterial DNA Quantification Kit (E2006, Zymo Research) according to the manufacturers’ instructions. Samples with a *C*_t_ value of more than 35 cycles or undetectable were counted as 0 pg ml^−1^.

### In vitro competition assay

The polyvinyl alcohol-based in vitro HSC expansion protocol was adapted as previously described^61,62^. 50 HSCs from wild-type GFP (C57BL/6-Tg(UBC-GFP)30Scha/J, 004353, Jackson Labs) and 50 HSCs from *Dnmt3a*^*−/−*^ mice were sorted directly into each well of a fibronectin-coated 96-well plate (08-774-60, Thermo Fisher Scientific) with Ham’s F12 nutrient mix medium (11765054, Thermo Fisher Scientific) containing final concentrations of 1× penicillin–streptomycin–glutamine (10378-016, Thermo Fisher Scientific), 10 mM HEPES (15630080, Thermo Fisher Scientific), 1× insulin–transferrin–selenium–ethanolamine (ITS-X, 51500056, Thermo Fisher Scientific), 100 ng ml^−1^ recombinant murine TPO (AF-315-14, Peprotech), 10 ng ml^−1^ recombinant murine SCF (250-03, Peprotech) and 1 mg ml^−1^ poly(vinyl alcohol) (P8136, Millipore Sigma) at 1:1 ratio at 37 °C and 5% CO_2_. Then, 1 µg ml^−1^ ADP-heptose treatment was started at day 8 after starting the culture when the second medium change was performed and added every 3 days with subsequent medium changes. After 14 days of ADP-heptose treatment, cells were collected, counted using the trypan blue exclusion assay and analysed by flow cytometry. To enumerate cells, a defined number of CountBright Absolute Counting Beads (Thermo Fisher Scientific, C36950) was added to each sample and cell count was back calculated to the proportion of the total that was run through the cytometer.

### Immunoblotting

For immunoblots, total protein lysates were obtained from cells by lysing the samples in cold RIPA buffer (50 mM Tris-HCl, 150 mM NaCl, 1 mM EDTA, 1% Triton X-100 and 0.1% SDS, in the presence of phenylmethylsulfonyl fluoride, sodium orthovanadate and protease and phosphatase inhibitors, as previously described^63^. After being resuspended in RIPA, cells were lysed by vortex followed by incubation on ice for 20 min. Protein concentration was evaluated using the bicinchoninic acid assay (Pierce, 23225). SDS sample buffer was added to the lysates and the proteins were separated by SDS–PAGE, transferred to PVDF or nitrocellulose membranes (Bio-Rad, 1620112) and analysed by immunoblotting. Western blot analysis was performed using the following antibodies: UBE2N (Abcam, ab25885; Cell Signaling, 6999 or 4919S, 1:1,000), vinculin (Cell Signaling, 13901T, 1:1,000), GAPDH (Cell Signaling, 5174T; D16H11, 1:1,000) phospho-IKKα/β (Ser176/180) (Cell Signaling, 2697, 1:1,000), MyD88 (Cell Signaling, 4283, 1:1,000), TRAF6 (Santa Cruz, sc-7221, 1:1,000), p65 (Cell Signaling, 8242, 1:1,000), phosphor-p65 (Ser536) (Cell Signaling, 3033, 1:1,000), IRAK4 (Cell Signaling, 4363, 1:1,000), IRAK1 (Santa Cruz, sc-5288, 1:1,000), phospho-SAPK/JNK (Thr183/Tyr185) (Cell Signaling, 4668, 1:1,000), SAPK/JNK (56G8) (Cell Signaling, 9258, 1:1,000), phospho-p38 MAPK (Thr180/Tyr182) (Cell Signaling, 4631, 1:1,000), p38 MAPK (Cell Signaling, 9212, 1:1,000), phospho-p44/42 MAPK (ERK1/2, Thr202/Tyr204) (Cell Signaling, 4377, 1:1,000), p44/42 MAPK (ERK1/2) (137F5) (Cell Signaling, 4695, 1:1,000), total IKKα/β (Cell Signaling, 2697, 1:1,000), ALPK1 (Abcam, ab236626), TIFA (Cell Signaling, 61358S, 1:1,000) and actin (Cell Signaling Technology, 4968, 1:1,000), and peroxidase-conjugated AffiniPure goat anti-rabbit IgG (Jackson ImmunoResearch Laboratories, 111-035-003, 1:10,000), and peroxidase-conjugated AffiniPure goat anti-mouse IgG (Jackson ImmunoResearch Laboratories, 115-035-003, 1:10,000). The membranes were visualized using ECL Western Blotting Substrate (Pierce, 32106) or SuperSignal West Femto Substrate (Thermo Fisher Scientific, 34096), imaged on the Bio-Rad ChemiDoc Touch Imaging system and analysed using Image lab software v.6.0.1 (Bio-Rad) or Image J (22930834).

### Quantitative analysis of TIFAsomes and ADP-heptose in biological samples using multispectral imaging flow cytometry (TIFAsome assay)

THP1 (THP1 *TIFA-TdTomato*) or THP1 *ALPK1*^*KO*^ cells (*ALPK1*^*KO*^*-TIFA-TdTomato* THP1) (1 × 10^6^) were stimulated with various human plasma samples (100 µl) for 30 min in a 37 °C water bath in a final volume of 200 µl. Cells were collected, washed with PBS + 2% FBS + 2 mM EDTA (MACS buffer) and fixed with 4% paraformaldehyde (15710, Electron Microscopy Sciences). After fixation, cells were washed again and then resuspended in 50 µl MACS buffer. Cells were then analysed for TIFAsome formation on the Amnis Imagestream Mk II Imaging Flow Cytometer ISX-100 (Luminex) according to the manufacturer’s instructions. Downstream analysis was performed using IDEAS analysis software (Amnis). TIFAsome-positive cells were identified by gating on the mean pixel intensity and maximum pixel intensity for bright puncta analysis using the IDEAS Image Data Exploration and Analysis Software. A standard curve was prepared by calculating the percentage of TIFAsome-positive cells using samples that were stimulated with serial increasing doses of ADP-heptose covering the concentration range of 10 to 100,000 ng ml^−1^. Using the data from the standard curve, the ADP-heptose concentration was extrapolated and estimated in unknown human biological samples using the following calculation: a standard curve was run, and the trend line was created. From the trendline equation *y* = *mx* + *b*, the concentration of ADP-heptose (*x*) was calculated by *x* = ((*y* − *b*)/*m*) × *d* where *m* is the slope of the trend line, *b* is the *y*-intercept, *x* is ADP-heptose concentration, *y* is percentage of positive TIFAsome cells and *d* is the dilution factor of the plasma.

To assess TIFAsome formation in mouse cells, HSPCs were purified from the BM of wild-type and knockout *Dnmt3a* mice using the CD117 MicroBeads, mouse (Miltenyi Biotech, 130-091-224) and cultured overnight in polyvinyl alcohol (PVA)-based medium on retronectin-coated plates. Cells were then transduced with pCDH-TIFA-TdTomato-GFP lentiviral particles and polybrene (0.8 µl ml^−1^) using the ultracentrifugation method. In brief, cells were centrifuged at 32 °C and 800*g* for 1.5 h and then cultured in fresh PVA medium for 3 days, after which KIT^+^GFP^+^TdTomato^+^ cells were sorted. Next, cells were stimulated with ADP-heptose (1 µg ml^−1^) and collected at serial timepoints (0 h, 0.5 h, 4 h, 24 h, 48 h), and subjected to TIFAsome assay as mentioned above.

### Immunofluorescence

*TIFA-TdTomato-GFP* THP1 cells were suspended at 1 × 10^6^ cells per ml and treated with either human plasma samples (50 µl) in final volume of 200 µl for 30 min or stimulated with ADP-heptose for 30 min. Cells were then washed and spun onto slides using a cytospin at 500 rpm at low acceleration. Slides were then fixed in PBS containing 4% paraformaldehyde and 0.1% Triton X-100. Slides were then blocked for non-specific binding in PBS with 3% bovine serum albumin and 0.1% Tween-20. The slides were mounted with ProLong Gold Antifade Mounting medium. Images were acquired using the Nikon Ni-E Upright widefield fluorescent scope and analysed using Nikon Elements.

### In vivo FITC–dextran permeability assay

As previously described^28,64^, the FITC assay is a measure of total intestinal permeability. In brief, mice were fasted for 5 h before the test at the beginning of the light cycle (12 h cycle) to minimize discomfort. When fasting, mice were transferred to a new cage (to limit coprophagy) without food or bedding but were kept with water bottles in the cage to avoid dehydration. After fasting, blood was collected by cheek bleeding on ice. Immediately after blood collection, the mice were then gavaged with freshly prepared 150 μl of 80 mg ml^−1^ fluorescently labelled smal-molecule FITC–dextran (4 kDa) (Sigma-Aldrich, 46944-500MG-F) diluted in sterile 1× PBS. Blood collection was repeated at 4 h after gavage and the mice were then returned immediately to their regular cages with bedding, food and water. Plasma was prepared by centrifugation of blood samples at 2,000*g* for 10 min at 4 °C and protected from light at all times. The FITC–dextran concentration in the plasma was measured using a fluorescence spectrophotometer with emission and excitation wavelengths of 520 nm and 490 nm, respectively.

### Haematological and histological analysis

Blood counts were measured using a Genesis blood analyzer (Oxford Scientific). Spleens, femurs and livers were fixed with 10% formalin, sectioned and stained with haematoxylin and eosin (H&E). Distal colonic tissues were fixed in 10% formalin, paraffin embedded and processed for H&E staining as previously described^65^. DSS-associated experimental colitis severity was assessed in a blinded manner by a pathologist using an established semi-quantitative multiparameter histopathological scoring system based on the following criteria: percentage area involved (0–4), oedema (0–3), ulceration (0–4), crypt loss (0–4) and leukocyte infiltration (0–3).

### Flow cytometry and cell sorting

Mice were euthanized using CO_2_ followed by cervical dislocation. PB was collected into EDTA-coated tubes (22030403, Thermo Fisher Scientific), and hind limb bones (femurs, and tibias) were obtained immediately after euthanasia and stored in cold FACS buffer (1% FBS in DPBS) under sterile conditions. Bones were crushed using a mortar and pestle and then passed through a 40 μm cell strainer (542040, Greiner Bio-one) for various applications. PB and BM cells were labelled with respective antibodies and analysed on a BD LSRII and BD Fortessa X-20 flow cytometers (BD Biosciences), and FACSDiva 8.0 and FlowJo software. For immunophenotypic analysis of PB samples, cells were first lysed with 1× red blood cell lysis buffer (555899, Thermo Fisher Scientific), and then incubated with CD19-PE (115507, BioLegend, 1:100), CD3-PerCpCy5.5 (100218, BioLegend, 1:100), Gr-1-APC (17-5931-81, eBioscience, 1:100) and CD11b-PE Cy5 (15-0112-82, eBioscience, 1:100). For HSPC analysis, BM cells were washed and incubated for 30 min with biotin-conjugated lineage markers (CD11b, Gr1, Ter119, CD3, B220, mouse haematopoietic lineage biotin panel (88–7774-75 eBioscience, 1:50)), followed by staining with streptavidin eFluor450 (48-4317-82, Thermo Fisher Scientific, 1:100), SCA1-PE (12–5981-82, eBioscience, 1:100), KIT-APC Cy7 (135135, BioLegend, 1:100), CD150-PerCp Cy5.5 (115922, BioLegend, 1:100) and CD48-APC (103412, BioLegend, 1:100). HSCs were identified on the basis of the expression of Lin^−^SCA1^+^KIT^+^CD150^+^CD48^−^. During in vitro HSC competition assay, to calculate the absolute number of cells whenever required, CountBright Absolute Counting Beads (C36950; Thermo Fisher Scientific, 1:10) were mixed with the cell sample (per well) and assayed by flow cytometry. By comparing the ratio of bead events to cell events, the absolute numbers of cells in the sample were calculated. For all of the experiments involving transplantations, to distinguish donor from recipient haematopoietic cells, PB and BM cells were also stained with CD45.1-Brilliant Violet 510 (110741, BioLegend, 1:100), and CD45.2-FITC (553772, Fisher Scientific, 1:100) or CD45.2-eFluor450 (48–0454-82, eBioscience, 1:100). HSCs were sorted as described previously^66^. BM cells were first enriched for stem/progenitor cells by using either lineage depletion (mouse total lineage kit, 130-110-470, Miltenyi Biotec, 1:50) or KIT enrichment kit (mouse CD117 microbeads, 130-091-224, Miltenyi Biotec, 1:50). KIT-enriched cells were immunostained for HSPC markers as mentioned above and sorted on the BD FACSAria II sorter (BD Biosciences).

### Measurement of cytokines and chemokines by multiplex ELISA

On day 1, wild-type, *Dnmt3a*^*−/−*^ and *Dnmt3a*^*−/−*^*Alpk1*^*−/−*^ mice were treated with ADP-heptose (0.5 mg per kg) twice (5 h apart). On day 2, mice were treated with ADP-heptose in the morning and the bones collected 5 h later. For preparing BM fluid, 2 femurs from each mouse were sectioned at the two ends and flushed with 200 µl of ice cold PBS containing 1× protease inhibitor (11836153001, Millipore Sigma), and transferred to cold Eppendorf tubes. After centrifugation at 1,000*g* for 5 min at 4 °C, the supernatant was immediately transferred to ice-cold Eppendorf tubes and frozen at −70 °C until further use. The samples were thawed on ice, vortexed thoroughly before being diluted 1:1 in assay buffer using the mouse cytokine/chemokine magnetic bead panel kit to quantify 32-plex mouse panel (MCYTOMAG-70K; Millipore Sigma).

### Colony forming cell assay

Clonogenic progenitor frequency was determined by plating freshly purified mouse LSK cells (5,000 cells per ml) in MethoCult GF M3434 (Stem Cell Technologies) or human patient samples (1,000 CD34^+^ cells per ml) in MethoCult H4434 (Stem Cell Technologies) in SmartDish meniscus-free 6-well plates in the presence of ADP-heptose (1 µg ml^−1^) with or without UBE2Ni (10 µM). Cells were incubated at 37 °C and 5% CO_2_. Colonies were scored at 14 days after plating using STEMVision (StemCell Technologies).

### Cell cycle analysis

As described previously^67^, mice were injected intraperitoneally with EdU (Invitrogen, 1 mg per mouse) and euthanized 6 h later and the EdU incorporation was analysed using the Click-iT Plus EdU Alexa Fluor 488 Flow Cytometry Assay Kit (C10633, Thermo Fisher Scientific).

### qPCR with reverse transcription

Total RNA was extracted and purified using the Quick-RNA MiniPrep (Zymo Research, R1055) or RNeasy Micro (Qiagen) kit, and reverse transcription was carried out using the Superscript complementary DNA Synthesis Kit (Invitrogen) or High-Capacity cDNA Reverse Transcription Kit (Thermo Fisher Scientific). qPCR was performed using the Taqman Master Mix (Life Technologies) for mouse *Alpk1* (Mm01320377_m1), *Tifa* (Mm07300088_m1) and *Gapdh* (Mm99999915_g1).

### Plasmids and viral transduction

As described elsewhere^34^, for generating N-terminal TIFA C-terminally fused to TdTomato, TIFA from human TIFA tagged ORF clone (NM_052864, RC204357, Origene) was amplified and cloned into pTdTomato N1 (54642, Addgene). For expressing TIFA-TdTomato fusion protein in THP1 cells, we cloned the corresponding *TIFA-TdTomato* cDNA into the pCDH-EF1-MCS-IRES-GFP vector plasmid from Systems Biosciences (CD530A-2). *TIFA-TdTomato* and pCDH vector were both incubated separately with EcoRI and NotI (New England Biosciences). Insert and vector fragments were run on a 1% agarose gel and extracted from the gel using a Qiagen Gel Extraction Kit (Qiagen). After gel extraction, insert and vector fragments were ligated together using T4 DNA ligase. DH5a competent cells were transformed with ligation products and streaked onto LB agar plates containing ampicillin for selection against negative clones. Colonies were picked, sequenced and a single correct clone was chosen for further study. Viral particles were produced using Mirus Trans-IT LT1 transfection reagent according to manufacturer protocols (Mirus). HEK293T cells were seeded to a confluency of approximately 80%. pCDH-TIFA-TdT-GFP plasmid was incubated with viral packaging plasmids containing gag-pol and VSV-G in the Trans-IT LT1 reagent (MIR 2306, Mirus). Packaged plasmid DNA was then added dropwise to seeded HEK293T. Cells were incubated for 48 h to allow for viral production. The viral supernatant was filtered and added to THP1 cells for transduction. Transduced cells were sorted for GFP^+^TdTomato^+^ using the BD FACSAria cell sorter, after which cells were grown in culture.

### Generation of mutant cells using CRISPR–Cas9 technology

THP1 *IRAK1*-knockout, THP1 *IRAK4*-knockout, THP1 *IRAK1/IRAK4*-double knockout, THP1 *MYD88*-knockout and THP1 *TRAF6*-knockout cells have been described previously^68^ THP1 *TIFA*-knockout cells were generated using a modified synthetic gRNA targeting exon 2 of the *TIFA* gene (Synthego)^3^ (sgRNA sequence, CAGAUGACGGUUUACCAUCC). THP1 *ALPK1*-knockout cells were generated using a synthetic multi-sgRNA kit targeting exon 5 of the *ALPK1* gene (Synthego gene knockout kit v2; human *ALPK1*). The sgRNAs used were as follows: sgRNA1, CAUCCUCGCUCGGGACUGUG; sgRNA 2, CUGUAUGGGCUCGACGUCUC; sgRNA3, AGUUCACGGAGAUUCGGGCU. Cells were generated by suspending the parental THP1 cells in buffer R with Cas9-NLS and sgRNA, and electroporated (1,700 mV × 20 ms × 1 pulse) using the Neon Transfection system (Invitrogen). As a control, THP1 *PTPRC*-knockout (*CD45*-knockout) cells were also generated using sgRNA targeting exon 2 of the *PTPRC* gene. The transfected cells were recovered for 48 h in antibiotic-free medium. *CD45* deletion was assessed by flow cytometry 5 days after transfection. Deletion for all other proteins was then confirmed by immunoblotting.

### In vivo pharmacokinetic analysis

Pharmacokinetic study of synthetic ADP-heptose was performed using our previously established protocol^53^ in C57BL/6 mice (18 to 22 g). ADP-heptose was administered through oral gavage (0.5 mg per kg per mouse). Blood samples were then collected at 4 h, 8 h, 16 h and 24 h after dosing. Samples from four animals were collected at each timepoint. About 200 µl of blood was collected through the orbital vein from each mouse, processed and analysed by LC–MS. A standard curve was prepared in blood covering the concentration range of 50 to 20,000 pg ml^−1^. Using the data from the standard curve, calibration curves were generated for pharmacokinetic tests.

### Bacterial culture of mouse tissues and human plasma

All of the mouse tissues were collected under sterile conditions with autoclaved tools by the same researcher throughout the study. In brief, PB was collected by cheek bleeding (after sterilizing the cheeks with 70% ethanol wipes) in sterile BD Microtainer Capillary Blood Collector and Microgard Closure tubes on ice from each mouse using Goldenrod Animal Lancets 4 mm. Plasma was then prepared by centrifuging at 5,000 rpm for 10 min. Bones (2 femurs and 2 tibiae per mouse) were grinded using a sterile mortar and pestle using sterile PBS. After red cell lysis using RBC lysis buffer (555899, BD Biosciences), the samples were resuspended in 300 µl of sterile filtered (0.22 μm) PBS + 0.1% l-cysteine (168149, Sigma-Aldrich) and homogenates prepared by homogenizing the samples in hard tissue homogenizing CK28 tubes (P000911-LYSK0-A, Bertin Instruments) using the Minilys homogenizer (Bertin Technologies). One fresh faecal pellet was collected from each mouse and resuspended in 1 ml of sterile PBS + 0.1% l-cysteine and homogenized in soil grinding SK38 tubes (P000915-LYSK0-A, Bertin Instruments) using the Minilys homogenizer. Then, 100 µl of mouse or human plasma, 100 µl of mouse BM homogenate and 100 µl of mouse faecal homogenates were then used for culturing on parafilm sealed Teknova brain heart infusion (BHI) agar plates (50-841-098, Thermo Fisher Scientific) and incubated upside down for 48 h. For all of the experiments, negative-control BHI plates were setup using 100 µl of sterile PBS + 0.1% l-cysteine; no colonies were observed in the negative control plates. Using sterile pipet tip, all of the colonies grown on BHI plates were scraped and mixed grown in 6 ml of liquid BHI broth for 24 h. Later, 750 µl of bacterial suspension was collected for immediate purification of bacterial DNA and remaining suspension used for preparation of lysates.

### RNA-seq analysis

Mouse LSK cells were purified from wild-type, *Dnmt3a*^*−/−*^ and *Dnmt3a*^*−/−*^*Alpk1*^*−/−*^ mice by flow cytometry and treated with ADP-heptose for 90 min in vitro. Total RNA was then extracted using the RNeasy Plus Micro Kit (Qiagen). The initial amplification step for all of the samples was performed using the NuGEN Ovation RNA-Seq System v.2. The assay was used to amplify RNA samples to create double-stranded cDNA. The concentrations were measured using the Qubit dsDNA BR assay. RNA libraries were then created for all samples using the Illumina protocol (Nextera XT DNA Sample Preparation Kit). The concentrations were measured using the Qubit dsDNA HS assay. The size of the libraries for each sample was measured using the Agilent HS DNA chip. The concentration of the pool was optimized to acquire at least 30–40 million reads per sample. The sequencing results were demultiplexed and converted to FASTQ format using Illumina bcl2fastq software. Paired-end FASTQ files were aligned to mm10 (mouse) genomes using HISAT2 (http://www.ccb.jhu.edu/software/hisat) or Tophat (https://ccb.jhu.edu/software/tophat). The feature Counts program (http://subread.sourceforge.net/)^69^ was used to generate counts for each gene based on how many aligned reads overlap its exons. These counts were then normalized and used to test for differential expression using negative binomial generalized linear models implemented by the DESeq2 R package (v.1.30.1). Further downstream analysis was performed with iGeak software^70^. We considered a gene as differentially expressed if statistically supported at FDR-adjusted *q* < 0.1 and a |log_2_[fold change]| > 0.5. Functional enrichment analysis was performed using the gene set enrichment analysis method^71^. All RNA-seq data generated in this study are available at the GEO (GSE232794).

### Xenograft and in vivo drug treatment

NOD.Cg-*Prkdc*^*scid*^*Il2rg*^*tm1Wjl*^*/*SzJ (NSG)^72^ mice were bred and maintained by the CCHMC Comprehensive Mouse Core. For patient-derived xenografts, NSG mice (sublethally conditioned with 2 Gy of whole-body irradiation) were injected into the tail vein with healthy CD34^+^ cells (1 × 10^6^ cells per mouse) and cells from individuals with MDS (5 × 10^6^ cells per mouse) in 200 µl of sterile PBS. Mice were then given sterile water or ADP-heptose (0.5 mg per kg) dissolved in sterile water at the indicated times. Mice were monitored for human engraftment in BM aspirates. In brief, 1 × 10^6^ BM cells from each sample were incubated with anti-human CD45 (555485, BD Biosciences, 1:100) and anti-human CD33 (555450, BD Biosciences, 1:100) antibodies in a solution of PBS, 0.2% FBS for 30 min on ice. Cells were washed once with PBS, resuspended in PBS with 0.2% FBS and immediately analysed by flow cytometry.

### NF-κB activation reporter

THP1-Blue NF-κB SEAP reporter cells (thp-nkfb, Invivogen) were grown at 20,000 cells per well (200 µl) in a 96-well plate with the indicated agonists and inhibitors for 24 h. The next day, QuantiBlue Reagent (Invivogen, rep-qbs2) was warmed to 37 °C in a water bath and 180 µl was added to each well of a new, clean 96-well plate. The incubated cells were centrifuged and 20 µl of supernatant from each well was pipetted into the respective 180 µl QuantiBlue Reagent well, in triplicate. The reaction was mixed and incubated for 1 h, when a colour gradient could be seen. The absorbance was read at 630 nm for a final readout. For analysis, the medium absorbance was subtracted, experimental values were normalized to the vehicle control and triplicates were averaged.

### Bacterial DNA extraction for 16S rRNA-seq

To examine the gut microbiota diversity and phylogenetics analysis, 16S rRNA-sequencing (rRNA-seq) was performed on faecal DNA isolated in a clean room environment from fresh faecal pellets using a previously described protocol^73^. In brief, faecal pellets were collected using sterile pipet tips in the biosafety cabinet from each mouse at the same time period of the day by same mouse handler throughout the study in sterile soil grinding SK38 tubes on ice. DNA was immediately extracted using the ultraclean QIAamp Fast DNA Stool Mini Kit (51604, Qiagen) according to the manufacturer’s recommendations using mechanical bead beating and a chemical-lysis-based approach to minimize kit contamination (kitome) and maintain low microbial biomass^74^. All of the mice were always housed in the same room. In a separate set of experiments, DNA was extracted from the bacterial suspension of various mouse tissues and human plasma using the same protocol. Ultraclean reagents were always used and all of the pre- and post-PCR and sequencing experiments were performed in separate designated area.

### Preparation of bacterial lysates for mass spectrometry

To measure ADP-heptose, lysates were prepared as described previously^44^. In brief, bacterial culture suspensions of various mouse tissues and human plasma samples were first centrifuged at 4,000*g* for 5 min. Next, the pellets were suspended in 1 ml of sterile water. Bacterial cells were then lysed by heating at 95 °C for 15 min. Next, the lysates were centrifuged at 4,000*g* for 3 min and the supernatants were filtered through a 0.20 µm syringe filter using 1 ml syringe and needle. Finally, lysates were stored at −80 °C until further processing for MS. According to a previously established protocol, the lysates were prepared^4^. In brief, 500 µl of the cleared bacterial lysate was thawed from −80 °C and extracted by addition of 1 ml chloroform:methanol (v/v, 2:1) and vortexed and centrifuged (5 min, 10,000*g*). The aqueous phase was then passed over the HyperSep solid phase extraction aminopropyl cartridge (200 mg per 3 ml) (60108-425, Thermo Fisher Scientific), which was initially equilibrated with 50 mM acetic acid in 50% methanol three times. Next, the bound compounds were eluted with 600 µl of 500 mM triethylammonium bicarbonate buffer (pH 8.5) in 50% methanol. Then, 600 μl of eluates was dried in the liquid N_2_ oxidation system with the lids open for 1.5 h. The samples were then solubilized in 1 ml of 10 mM ammonium bicarbonate (pH 8.0), vortexed and passed over the graphite carbon Supelclean Envi-Carb 1 ml column (57109-U, Millipore Sigma) which was pre-equilibrated with 80% acetonitrile + 0.1% formic acid. Next, the bound compounds were eluted with 800 µl of 30% acetonitrile + 10 mM ammonium bicarbonate. Eluate was dried in the liquid N_2_ oxidation system with the lids open for 2 h. The dried pellets were then frozen in −20 °C.

### UHPLC–MS/MS analysis

The concentration of ADP-heptose was determined using an ultra-high-performance liquid chromatography–electrospray ionization MS (UHPLC–ESI-MS/MS) method by modifying our previously described protocol^75^. A linear calibration curve was generated in the range of 1–1,000 ng ml^−1^ and ^13^C_6_-UDP-glucose (CLM-10513-0.001; Cambridge Isotope Laboratories) was used as an internal standard throughout the assay. The internal standard (10 µl of a 50 ng µl^−1^ methanol solution) was added to the bacterial lysate samples and to the calibrators and quality-control samples. The 5 µl volume of sample extract was injected on column for analysis by electrospray ionization UHPLC–MS/MS using a Waters TQ-XS triple quadruple mass spectrometer interfaced with an Equity UPLC system. The optimal signals for the ion pair of analyte and internal standard, that is, *m*/*z* 617.8 to 270.7 for ADP-heptose and *m*/*z* 570.6 to 322.7 for ^13^C_6_-UDP-glucose, respectively, were achieved in negative-ion mode with the use of the following instrument settings: capillary voltage, 3.0 kV; source temperature, 120 °C; desolvation temperature, 350 °C; desolvation gas flow, 800 l h^−1^; and cone gas flow, 150 l h^−1^. The cone voltage, collision energy and ion dwell time were optimized and were set at 30 V, 30 ev, 0.1 s respectively; helium was used as the collision gas. An ACQUITY UPLC BEH Amide column (2.1 mm × 100 mm, 1.7 µm) was used in separation. A gradient mobile phase was used with a binary solvent system, which started with 25% solvent A and was held for 1 min, changed from 25% solvent A to 100% solvent A over 4 min, held for 2 min, then to 25% solvent A at 7.1 min, and this was held for 3 min. The total run time was 10 min, and the flow rate was 0.2 ml min^−1^. Solvent A consisted of acetonitrile:water (5:95) with 20 mM ammonium acetate and was adjusted to pH 9.5 with ammonium hydroxide; solvent B consisted of acetonitrile. The injection volume was 5 μl. Data were acquired and processed using Masslynx v.4.1 (Waters).

### Faecal microbiota transplantation

To prepare faecal material for transplantation, previously established protocols were followed^76,77^, and the samples were processed within 30 min of collection. Fresh faecal samples (6 pellets per mouse) were collected from either H_2_O-treated or DSS-treated mice in 2 ml homogenizer-compatible tubes in 1.5 ml of sterile PBS containing 0.05% l-cysteine HCl reducing agent to preserve anaerobes. After vigorous homogenization at high speed twice to confirm proper mixing, the faecal suspension was filtered through a 40 µM cell strainer to clear away the particulate matter. To further clear out undissolved solids matter and concentrate bacteria, the tubes were centrifuged at 800*g* for 3 min at 4 °C and the supernatant was collected and diluted with 4.5 ml transfer buffer (1:3). Then, 1 ml aliquots were prepared, stored in 10% glycerol at −80 °C, and used for experiment within 2 weeks of collection. Chimeric mice, as mentioned before, were pretreated with antibiotics cocktail for 4 weeks and then transplanted with faecal material for 4 weeks (twice a week oral gastric gavage 100 µl; every Tuesday and Thursday) after which flow cytometry was performed on BM. To confirm whether faecal microbial transplantation was successful, faecal samples were collected from the donor (H_2_O- and DSS-treated wild-type) and recipient (*Dnmt3a* wild-type and knockout) mice at serial timepoints after transplantation (days 3, 7 and 14). DNA was extracted and 16S rRNA-seq was performed to analyse the taxonomic classification of the colonized bacteria.

### 16S rRNA-seq and analysis

16S rRNA library preparation and metagenomic sequencing was performed using a previously defined protocol. Sequencing was performed using the primer set, 515F (GTGCCAGCMGCCGCGGTAA) and 806R (GGACTACHVGGGTWTCTAAT), which covers the V4 region to run as PE250 on the MiSeq platform. Read pairs from the raw sequencing data were demultiplexed based on barcodes and downstream data processes were done using USEARCH/UPARSE v.11.0.667_i86linux32 (https://www.drive5.com/usearch)^78^. In brief, forward and reverse reads were first merged using -fastq_merge pairs, primers were striped (-stripleft 19 -stripright 20) using -fastx_truncate. As low-quality reads often cause spurious OUTs, reads were filtered using -fastq_filter to discard reads with expected error scores below 1. After filtering, the reads were dereplicated with -fastx_uniques. Unique reads were used as input for the uparse step, using -cluster_otus. The -cluster_otus command performs 97% operational taxonomic unit (OTU) clustering, and removes chimeric sequences. The resulting OTU table was normalized to 5,000 reads using -otutab_norm. The OTU tree was also generated using -otutab_norm and -cluster_agg commands. Error-corrected reads (ZOTU, denoised OTU) were identified using the denoising step (-unoise2 command) For taxonomic classification of the bacterial ZOTUs, the -sintax command was used with the reference training set RDP training set v18 (rdp_16s_v18, https://www.drive5.com/usearch/manual/sintax_downloads.html). All non-bacterial ZOTUs were removed on the basis of their taxonomic classification. Microbiome communities in comparison groups were analysed using the R package phyloseq (https://joey711.github.io/phyloseq/). The ZOTU table, the ZOTU taxonomy, the ZOTU tree and the sample table were imported into phyloseq to create the phyloseq object. The possible contaminating DNA features were statistically identified using the decontam package (https://bioconductor.org/packages/release/bioc/html/decontam.html) and the sequenced DNA concentration (efficient ng μl^−1^) data for each sample. Samples showing signs of substantial contamination were removed at this stage. Statistical analysis of the number of reads, length and mean quality (phred) score were verified using FastQC (v.0.11.8). Example quality scores across the entire read length are presented in Supplementary Table 16. A score of >30 is considered to be very good quality 16S sequence reads. The mean number of reads across all human samples (*n* = 32) was 131,706 with mean phred *Q* score of 35.8 and, across mouse samples (*n* = 78), the mean read depth was 188,177 with mean *Q* score of 35.7. Any sample with normalized ZOTU < 5,000 reads was not included in the final data analysis. Alpha diversity metrics were computed using the R package vegan (functions diversity, estimate and spec number for Shannon indicator, Chao1 index and observed richness, respectively). Taxonomic classification was then finally investigated. All of the 16S rRNA-seq data generated in this study are provided in Supplementary Tables 17–20 and are available under BioProject ID PRJNA1055136.

### Publicly available datasets

RNA-seq data of individuals with AML^79^ were downloaded from the GDC Data Portal (https://portal.gdc.cancer.gov/) and the BEAT AML (Vizome, http://www.vizome.org/aml/)^80^. Published microarray data of individuals with MDS^81^ and respective age-matched controls were downloaded from the GEO (GSE58831). DNA methylation data of wild-type and *Dnmt3a*^*−/−*^ HSCs were obtained from the GEO (GSE98191)^23^.

### Statistical analysis

No statistical methods were used to predetermine sample size. The number of animals, cells and experimental/biological replicates can be found in the figure legends. Differences among multiple groups were assessed using one-way and two-way ANOVA followed by multiple-comparison post-testing for all possible combinations. Comparison of two groups was performed using Mann–Whitney *U*-tests or Student’s *t*-tests (unpaired, two-tailed) when the sample size allowed. Unless otherwise specified, the results are depicted as the mean ± s.d. or mean ± s.e.m. A normal distribution of data was assessed for datasets > 30. For correlation analysis, the Pearson correlation coefficient (*r*) was calculated. For Kaplan–Meier analysis, Mante–Cox tests were used. All graphs and analysis were generated using GraphPad Prism 9.0 software or using the package ggplot2 from R. For all analyses, *P* < 0.05 was considered to be statistically significant. Investigators were not blinded to the different groups. We used the publicly available relative-risk calculator (https://www.gigacalculator.com/calculators/relative-risk-calculator.php) to compute the relative risk (risk ratio), confidence intervals and *P* values for risk assessment between individuals with CHIP/clonal cytopenias of unknown significance with ADP-heptose present and those with ADP-heptose absent in circulation.

### Reporting summary

Further information on research design is available in the Nature Portfolio Reporting Summary linked to this article.

## Online content

Any methods, additional references, Nature Portfolio reporting summaries, source data, extended data, supplementary information, acknowledgements, peer review information; details of author contributions and competing interests; and statements of data and code availability are available at 10.1038/s41586-025-08938-8.

## Supplementary information

Supplementary Figs. 1 and 2 Supplementary Fig. 1: uncropped western blot images for Fig. 3 and Extended Data Figs. 7, 8 and 10. Supplementary Fig. 2: representative gating schemes for flow cytometry.

Reporting Summary

Supplementary Tables Supplementary Tables 1–20.

## Source data

Source Data Figs. 1–3 and 5 and Source Data Extended Data Figs 1–10.

## Data Availability

RNA-seq data generated in this study have been deposited at the NCBI Gene Expression Omnibus (GEO) repository under accession number GSE232794. 16S rRNA-seq data generated in this study have been deposited at BioProject (PRJNA1055136). The mouse reference genome mm10 was used for mapping the reads. RNA-seq data of individuals with AML were downloaded from the GDC Data Portal (https://portal.gdc.cancer.gov/) and BEAT-AML (Vizome; http://www.vizome.org/aml/). Published microarray data of individuals with MDS, and respective age-matched controls were downloaded from GSE58831. DNA methylation data of *Dnmt3a*^*+/+*^ and *Dnmt3a*^*−/−*^ HSCs were obtained from GSE9819137. All individual mouse lines used in this study are commercially available at The Jackson Laboratory. Plasmid constructs and cell lines used in this study are available from the corresponding author on request. Source data are provided with this paper.
