# Supplementary material for: Microbial metabolite drives ageing-related clonal haematopoiesis via ALPK1
Source: Nature. 2025 Apr 23;642(8066):201–11. doi: 10.1038/s41586-025-08938-8 (PMC12137129; doi:10.1038/s41586-025-08938-8)
Supplement: Supplementary file 1 — Supplementary Fig. 1: uncropped western blot images for Fig. 3 and Extended Data Figs. 7, 8 and 10. Supplementary Fig. 2: representative gating schemes for flow cytometry. [file 41586_2025_8938_MOESM1_ESM.pdf]

nature portfolio

https://doi.org/10.1038/s41586-025-08938-8

Supplementary information

Microbial metabolite drives ageing-related clonal haematopoiesis via ALPK1

In the format provided by the authors and unedited

Supplementary Fig. 1

Fig. 3i

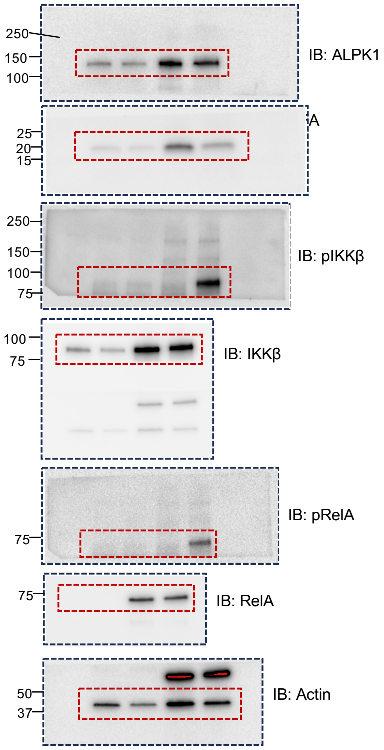

Fig. 3p

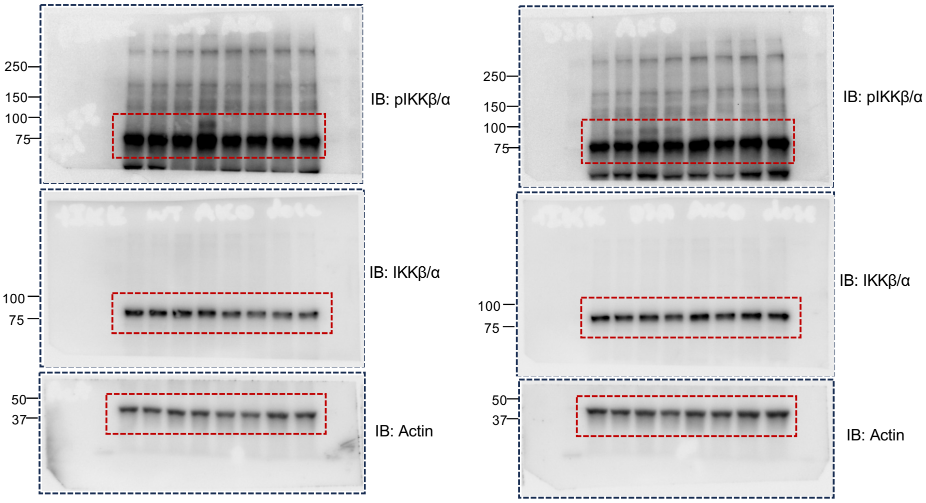

Extended Data Fig. 7d

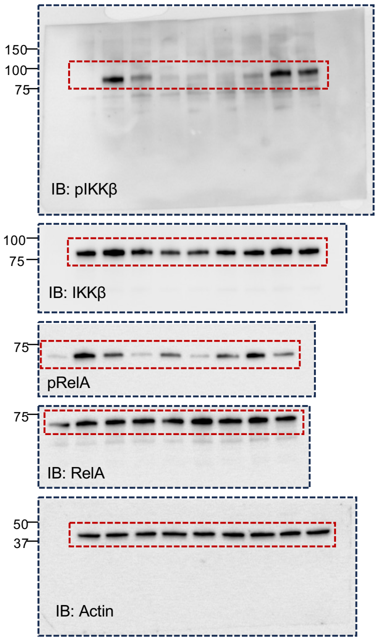

Extended Data Fig. 7f

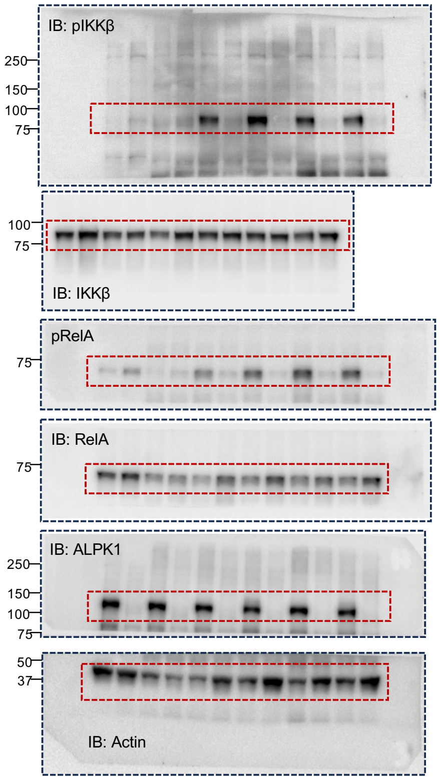

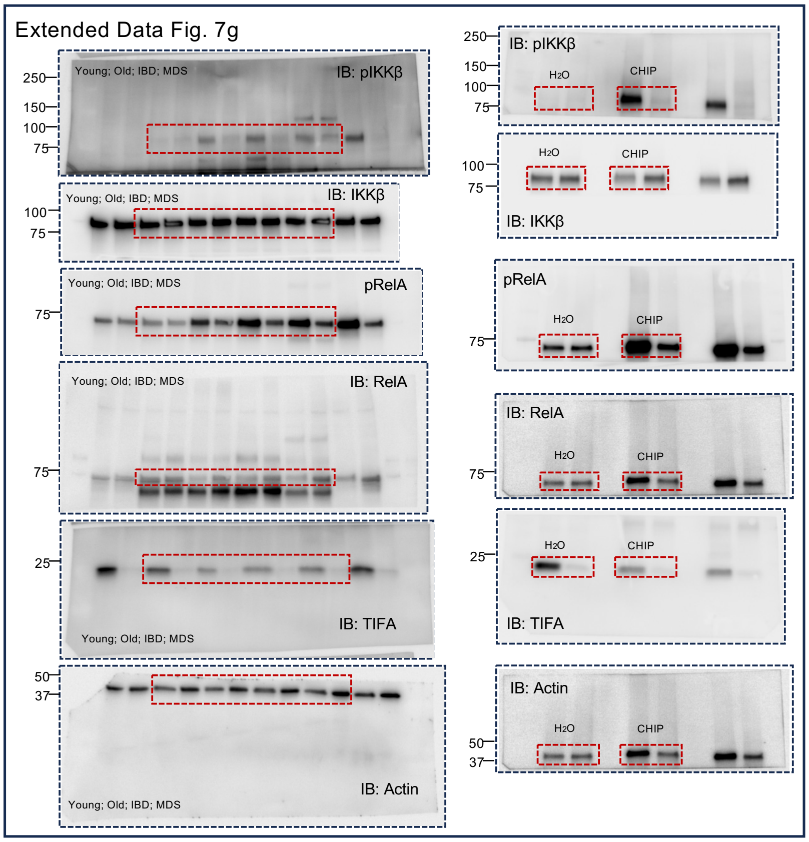
Extended Data Fig. 7g
IB: pIKKβ
Young; Old; IBD; MDS
IB: IKKβ
pRelA
IB: RelA
IB: TIFA
IB: Actin
H2O
CHIP
250
150
100
75
25
50
37

Extended Data Fig. 7h

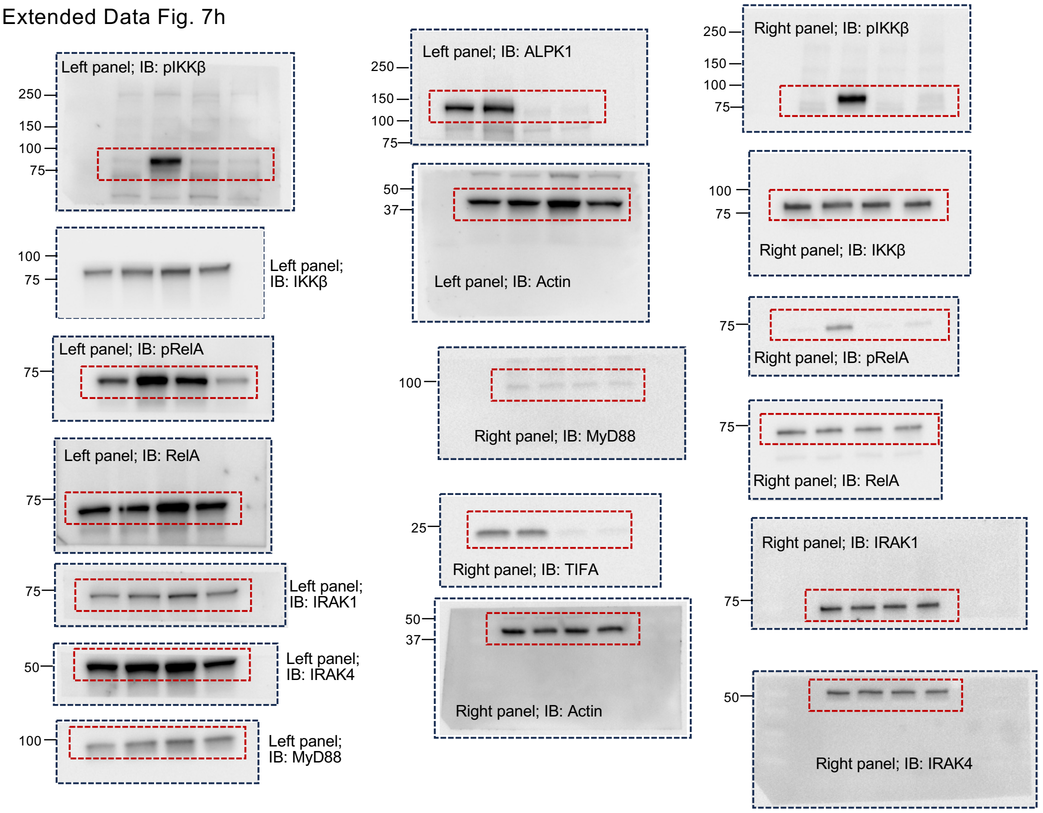

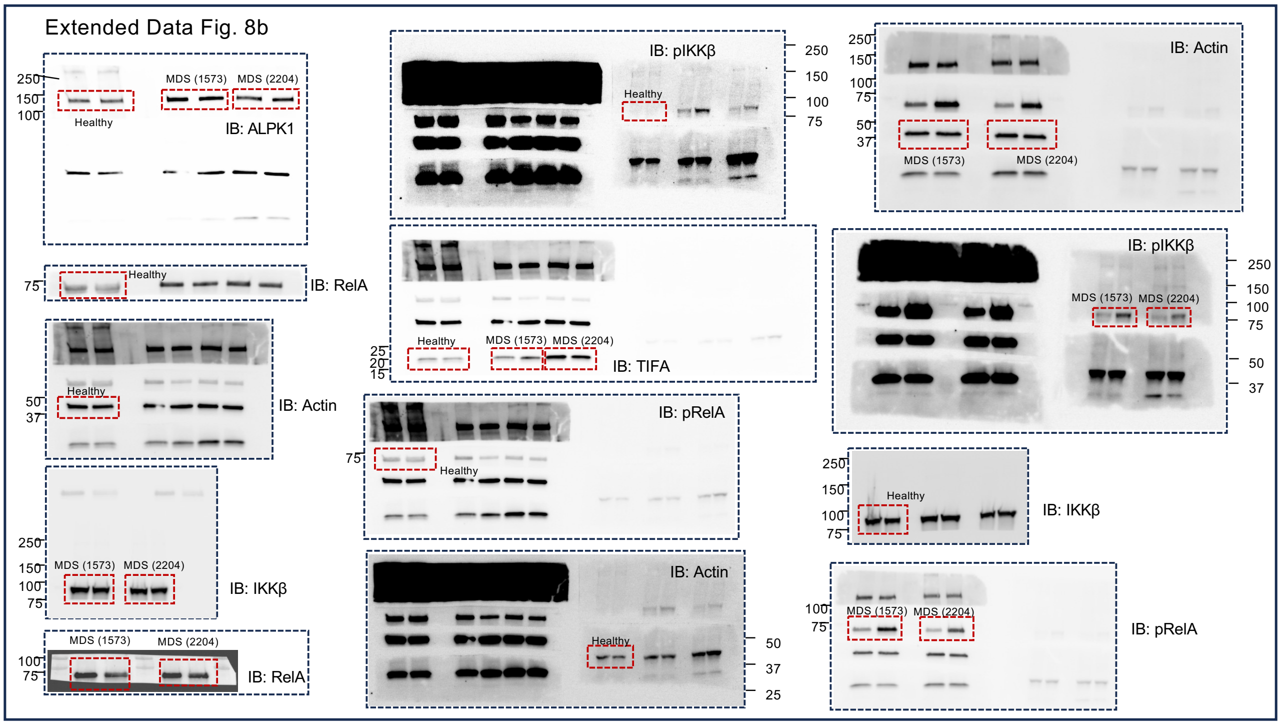
Extended Data Fig. 8b
IB: ALPK1
Healthy
MDS (1573)
MDS (2204)
250
150
100
IB: RelA
75
IB: Actin
50
37
IB: IKKβ
IB: pIKKβ
IB: TIFA
IB: pRelA
25
20
15

Extended Data Fig. 8f

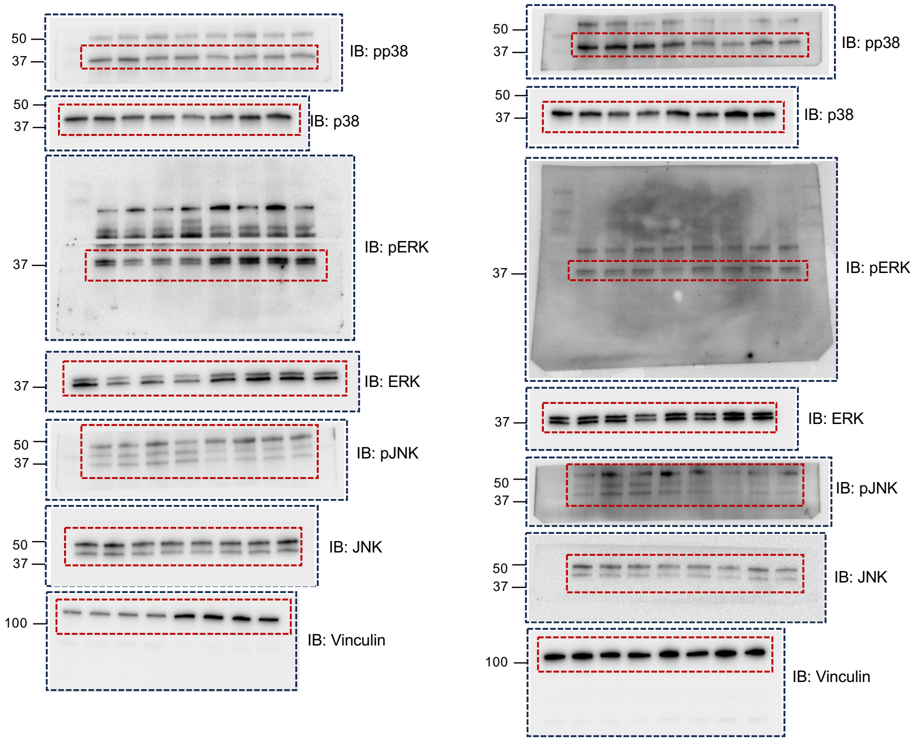

Extended Data Fig. 10e

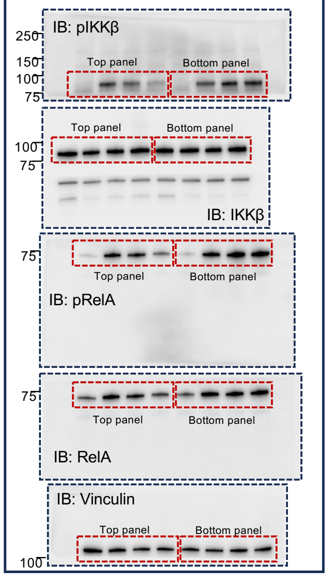

Extended Data Fig. 10f

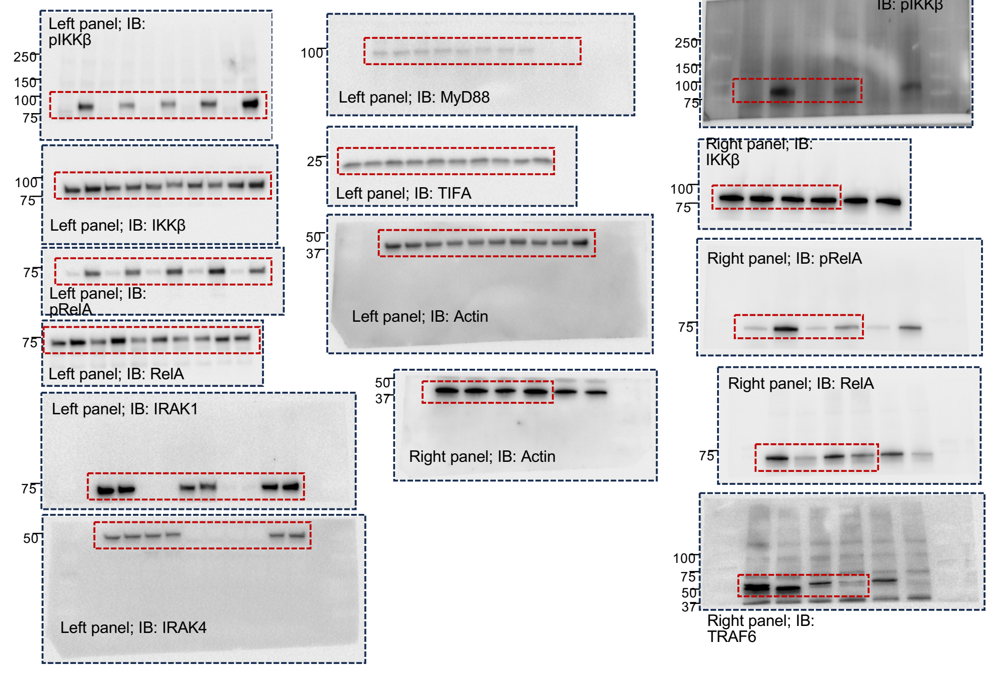

Supplementary Fig. 2

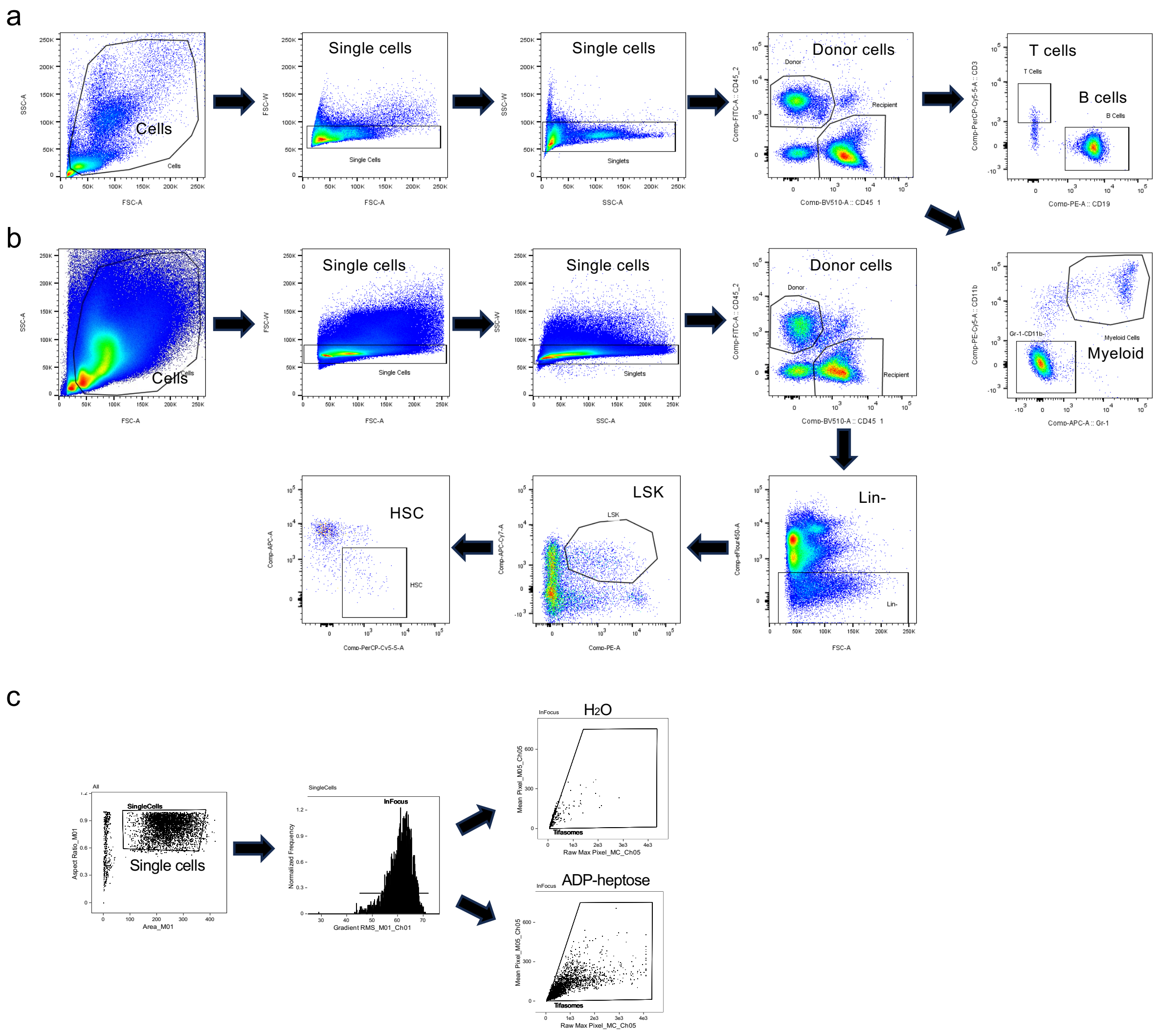

Representative gating strategies for all FACS isolation and flow cytometric analyses performed in the study. **a,** Gating strategy to analyze donor-derived multilineage chimerism in peripheral blood and bone marrow (Fig. 1c, 2f, 3r; Extended Data Fig. 1i, 1j, 2d, 3c, 5b, 5e, 5f, 5i, 5j, 9c, 9g). **b,** Gating strategy to examine and sort donor-derived hematopoietic stem cells (HSCs) in the BM (Fig. 1b,1e,1i, 2e, 3q, 5a; Extended Data Fig. 1h, 2e, 3b, 4b, 4d, 5d, 5h, 9b). **c,** Gating strategy to examine TIFAsomes on ImageStream. Shown are THP1-TIFA-TdTomato cells treated with H_2O (Top panel) or ADP-heptose (1 μg/ml) (Bottom panel) for 30 mins. (Fig. 3a, 3b, 3c, 3d, 3e, 3f, 3n; Extended Data Fig. 6a, 6b, 6c, 6d, 6e, 7a, 7b, 7c).
