# Supplementary material for: Microbial metabolite drives ageing-related clonal haematopoiesis via ALPK1
Source: Nature. 2025 Apr 23;642(8066):201–11. doi: 10.1038/s41586-025-08938-8 (PMC12137129; doi:10.1038/s41586-025-08938-8)
Supplement: Supplementary file 2 — Reporting Summary [file 41586_2025_8938_MOESM2_ESM.pdf]

nature portfolio

Corresponding author(s): Daniel Starczynowski

Last updated by author(s): Mar 25, 2025

Reporting Summary

Nature Portfolio wishes to improve the reproducibility of the work that we publish. This form provides structure for consistency and transparency in reporting. For further information on Nature Portfolio policies, see our Editorial Policies and the Editorial Policy Checklist.

Statistics

For all statistical analyses, confirm that the following items are present in the figure legend, table legend, main text, or Methods section.

n/a	Confirmed	
☐	☒	The exact sample size (*n*) for each experimental group/condition, given as a discrete number and unit of measurement
☐	☒	A statement on whether measurements were taken from distinct samples or whether the same sample was measured repeatedly
☐	☒	The statistical test(s) used AND whether they are one- or two-sided<br>*Only common tests should be described solely by name; describe more complex techniques in the Methods section.*
☒	☐	A description of all covariates tested
☒	☐	A description of any assumptions or corrections, such as tests of normality and adjustment for multiple comparisons
☒	☐	A full description of the statistical parameters including central tendency (e.g. means) or other basic estimates (e.g. regression coefficient) AND variation (e.g. standard deviation) or associated estimates of uncertainty (e.g. confidence intervals)
☐	☒	For null hypothesis testing, the test statistic (e.g. *F*, *t*, *r*) with confidence intervals, effect sizes, degrees of freedom and *P* value noted<br>*Give P values as exact values whenever suitable.*
☒	☐	For Bayesian analysis, information on the choice of priors and Markov chain Monte Carlo settings
☒	☐	For hierarchical and complex designs, identification of the appropriate level for tests and full reporting of outcomes
☐	☒	Estimates of effect sizes (e.g. Cohen's *d*, Pearson's *r*), indicating how they were calculated

Our web collection on statistics for biologists contains articles on many of the points above.

Software and code

Policy information about availability of computer code

Data collection	All the FACS data was collected with FACSdiva 8.0 (BD Bioscience). Colony formation assay images were captured using STEMvision Stem Cell Technologies. Immunoblots and nucleic acid gels were captured using Image Lab 6.1 (BioRad). No custom scripts were used to collect data for this study.
Data analysis	Numerical data was first processed with Microsoft Excel 2016 and then analyzed and plotted with Graphpad Prism v9. All FACS data were analyzed with FlowJo 10.8 and FACSDiva on PC. All p-values and graphs were generated with GraphPad Prism 9 and the ggplot2 package from R. Publicly available relative risk calculator (https://www.gigacalculator.com/calculators/relative-risk-calculator.php) was used to compute the relative risk (risk ratio), confidence intervals and p values for risk assessment. We used Image J software (1.46R) to quantify DNA and protein band intensity. The mass spectrometry data were acquired and processed with Masslynx 4.1 software (Waters). Bulk RNA-sequencing results were demultiplexed and converted to FASTQ format using Illumina bcl2fastq software. Paired-end FASTQ files were aligned to mm10 (mouse) genomes using HISAT2 (version 2.0.0-beta) (http://www.ccb.jhu.edu/software/hisat) or Tophat (https://ccb.jhu.edu/software/tophat). The feature Counts program (http://subread.sourceforge.net/)11 was utilized to generate counts for each gene based on how many aligned reads overlap its exons. These counts were then normalized and used to test for differential expression using negative binomial generalized linear models implemented by the DESeq2 R package (v.1.30.1). Further downstream analysis was performed with iGeak software (Choi et al. 2019; PMID: 30841853). Functional enrichment analysis was performed using the gene set enrichment analysis (GSEA) method. Adobe Illustrator CC v27.5 were used for downstream image analysis and illustration. Some of the figures in the manuscript were created using BioRender (https://www.biorender.com/).<br>For 16S rRNA analysis, sequencing was performed on the MiSeq platform. Read pairs from the raw sequencing data were de-multiplexed based on barcodes and downstream data processes were done using USEARCH/UPARSE v11.0.667_i86linux32 (https://www.drive5.com/usearch). For taxonomic classification of bacterial ZOTUs, the reference training set RDP training set v18 (rdp_16s_v18, https://www.drive5.com/usearch/manual/sintax_downloads.html). Microbiome communities were analyzed using the R package phyloseq (https://

joey711.github.io/phyloseq/). Possible contaminating DNA features were statistically identified using the decontam package (https://bioconductor.org/packages/release/bioc/html/decontam.html). Statistics of the number of reads, length, and mean quality (phred) score were verified using FastQC (v.0.11.8). Example quality scores across the entire read length are presented in Supplementary Table16. Alpha diversity metrics were computed using the R package vegan (functions diversity, estimate and spec number for Shannon indicator, Chao1 index and observed richness, respectively).

For manuscripts utilizing custom algorithms or software that are central to the research but not yet described in published literature, software must be made available to editors and reviewers. We strongly encourage code deposition in a community repository (e.g. GitHub). See the Nature Portfolio guidelines for submitting code & software for further information.

Data

Policy information about availability of data

All manuscripts must include a data availability statement. This statement should provide the following information, where applicable:

- Accession codes, unique identifiers, or web links for publicly available datasets
- A description of any restrictions on data availability
- For clinical datasets or third party data, please ensure that the statement adheres to our policy

All RNA sequencing and 16S rRNA sequencing data generated in this study have been deposited at NCBI's GEO repository with accession number's GSE232794 and BioProject ID: PRJNA1055136, respectively. Mus musculus mm10 genome was used for annotation. RNA-sequencing data of AML patients were downloaded from the GDC Data Portal (https://portal.gdc.cancer.gov/) and BEAT-AML (Vizome, http://www.vizome.org/aml/). Published microarray data of patients with MDS, and respective age matched controls were downloaded from GSE58831. DNA methylation data of Dnmt3a+/+ and Dnmt3a-/- HSCs was obtained from GSE9819137. Plasmid constructs and cell lines used in this study are available from the corresponding author upon request. Source data are provided with this paper.

Research involving human participants, their data, or biological material

Policy information about studies with human participants or human data. See also policy information about sex, gender (identity/presentation), and sexual orientation and race, ethnicity and racism.

Reporting on sex and gender	Plasma was obtained from both males and females with diverse race and ethnicities. Plasma from young and aged healthy individuals, individuals diagnosed with IBD or MDS were obtained from BioIVT. For detailed information, refer Supplementary Table 3 and 4.
Reporting on race, ethnicity, or other socially relevant groupings	N/A.
Population characteristics	All the relevant clinical characteristics of patients such as age, sex, race, ethnicity, past and current diagnosis, treatment history, other co-morbidities, etc. are provided in Supplementary Table 4.
Recruitment	Human plasma samples were obtained from multiple sources. Plasma from healthy individuals (young [<65 years], n = 5; old [≥65 years], n = 10), and individuals diagnosed with IBD (n = 8) or MDS (n = 9) were obtained from BioIVT. IBD and MDS patients with secondary malignancy (AML) were excluded. IBD patients with prior treatment were excluded. Plasma from MDS (n = 20) and AML (n = 15) individuals was obtained from Ohio State University. MDS patients with secondary malignancy (AML) were excluded. Plasma from healthy individuals (young [<65 years], n = 6; old [≥65 years], n = 7), IBD (n = 3), and CHIP (n = 29) were obtained from subjects undergoing elective total hip replacement surgery under the Mechanisms of Age-Related Clonal Haematopoiesis (MARCH) Study (NHS REC Ref: 17/YH/0382) at the Oxford University Hospital, UK. IBD patients with prior treatment were excluded. Plasma from CHIP individuals (n = 30) was obtained from the University of Cincinnati. All participants gave written informed consent in accordance with the Declaration of Helsinki. For detailed information, refer to Supplementary Table 4 and 5.
Ethics oversight	Oxford University Hospital, UK; and University of Cincinnati Hospital, Cincinnati, USA

Note that full information on the approval of the study protocol must also be provided in the manuscript.

Field-specific reporting

Please select the one below that is the best fit for your research. If you are not sure, read the appropriate sections before making your selection.

☒ Life sciences ☐ Behavioural & social sciences ☐ Ecological, evolutionary & environmental sciences

For a reference copy of the document with all sections, see nature.com/documents/nr-reporting-summary-flat.pdf

Life sciences study design

All studies must disclose on these points even when the disclosure is negative.

Sample size	For in vitro experiments, sample size calculation was based on power analysis and historical observations to detect a >2-fold increase (student's t-test, $p < 0.05$). Hence, at least 3 biological replicates were performed/utilized.<br>For in vivo experiments involving mice, treatment group size was estimated based on biostatics consultation, as well as historical

observations. In order to reduce the number of experimental animals used, experiments were planned in an effort to provide 60%-80% power for a target effect size of 1.2-1.5 (effect size=|mean difference|/SD).

Data exclusions	No data were excluded.
Replication	All mouse experiments used both male and female mice. Based on our extensive experience, xenograft experiments were performed using >5 recipients per condition to detect 65% relative treatment differences with 80% power at a significance level of 0.05. All in vitro/ex vivo experiments have both biological and technical replicates and be repeated multiple times. In the manuscript, n represents the number of samples/animals per experiment (each experiment being representative of at least three independent experiments). All results are expressed as means with error bars reflecting standard error of the mean (unless otherwise specified). Differences between two groups were assessed using unpaired two-tailed Student's t-tests. To ensure proper tests were performed (e.g., Student's t-test, one-way ANOVA, two-way ANOVA, log-rank tests, etc.), a full-time statistician within our division was consulted. Throughout the study, all the experiments involved replication in at least three independent experiments to ensure reproducibility.
Randomization	For in vivo experiments involving mice, animals of the same age and gender were randomly assigned to experimental groups. For in vitro experiments, it was critical to know the cell lines before conducting the experiments for data collection and data analyses. No hypothesis was tested regarding molecular entities. All samples were treated equally using the same rigorous criteria to avoid bias.
Blinding	No blinding was performed, as genotyping and treatments were necessary for data collection and analyses of all the experiments.

Reporting for specific materials, systems and methods

We require information from authors about some types of materials, experimental systems and methods used in many studies. Here, indicate whether each material, system or method listed is relevant to your study. If you are not sure if a list item applies to your research, read the appropriate section before selecting a response.

Materials & experimental systems

n/a	Involved in the study	
☐	☒	Antibodies
☐	☒	Eukaryotic cell lines
☒	☐	Palaeontology and archaeology
☐	☒	Animals and other organisms
☒	☐	Clinical data
☒	☐	Dual use research of concern
☒	☐	Plants

Methods

n/a	Involved in the study	
☒	☐	ChIP-seq
☐	☒	Flow cytometry
☒	☐	MRI-based neuroimaging

Antibodies

Antibodies used

For western blotting:
UBE2N (Abcam, ab25885; Cell Signaling, #6999 or #4919S)
Vinculin (Cell Signaling, #13901T)
GAPDH (Cell Signaling, #5174T; D16H11)
phospho-IKKα/β (Ser176/180) (Cell Signaling, #2697)
MyD88 (Cell Signaling, #4283)
TRAF6 (Santa Cruz, #sc-7221)
p65 (Cell Signaling, #8242)
phosphor-p65 (Ser536) (Cell Signaling, #3033)
IRAK4 (Cell Signaling, #4363)
IRAK1 (Santa Cruz, #sc-5288)
phospho-SAPK/JNK (Thr183/Tyr185) (Cell Signaling, #4668)
SAPK/JNK (56G8) (Cell Signaling, #9258)
phospho-p38 MAPK (Thr180/Tyr182) (Cell Signaling, #4631)
p38 MAPK (Cell Signaling, #9212)
phospho-p44/42 MAPK (ERK1/2. Thr202/Tyr204) (Cell signaling, #4377)
p44/42 MAPK (Erk1/2) (137F5) (Cell Signaling, #4695)
Total-IKKα/β (Cell Signaling, #2697)
ALPK1 (MyBioSource, #MBS001969)
TIFA (Cell Signaling, #61358S)
Actin (Cell Signaling Technology, #4968)
Peroxidase-conjugated AffiniPure goat anti-rabbit IgG (Jackson ImmunoResearch Laboratories, #111-035-003)
Peroxidase-conjugated AffiniPure goat anti-mouse IgG (Jackson ImmunoResearch Laboratories, #115-035-003).

For flow cytometry:
CD33-PE (#555450, BD Biosciences)
CD45-APC (#555485, BD Biosciences)
CD19-PE (#115507, BioLegend)
CD3-PerCpCy5.5 (#100218, BioLegend)
Gr-1-APC (#17-5931-81, eBioscience)

CD11b-PE Cy5 (#15-0112-82, eBioscience)
Lineage biotin panel (#88–7774-75 eBioscience)
Streptavidin-eFluor450 (#48-4317-82, Thermo Fisher Scientific)
Sca-1-PE (#12–5981-82, eBioscience)
c-Kit-APC Cy7 (#135135, BioLegend)
Flk2-PE-Cy5 (#15–1351-81, eBioscience)
CD150-PerCp Cy5.5 (#115922, BioLegend)
CD48-APC (#103412, BioLegend)
CD45.1-Brilliant Violet 510 (110741, BioLegend)
CD45.2-Fitc (553772, Fisher Scientific)
CD45.2-eFluor450 (48–0454-82, eBioscience)
Mouse Lineage depletion kit (#130-110-470, Miltenyi Biotec)
Mouse c-Kit enrichment kit (#130-091-224, Miltenyi Biotec)

Validation	All western blot antibodies used have been previously reported and validated for use in the relevant species by the corresponding manufacturer, which is described in the manufacturer's website except for ALPK1 (MyBioSource, #MBS001969) which was validated in this study. All flow cytometry antibodies were previously reported and validated by comparing their staining pattern on BM cells by FACS with appropriate negative and positive controls, and also previously reported and validated for use in the relevant species by the corresponding manufacturer, which is described in the manufacturer's website. The validation statements and published references are on the manufacturer's websites. Our usage is described in the Methods section of the manuscript.

Eukaryotic cell lines

Policy information about cell lines and Sex and Gender in Research

Cell line source(s)	THP1 cells (derived from male AML patient) were purchased from American Type Culture Collection (ATCC, #TIB-202).<br>HEK293T cells (derived from the kidney of a patient) were purchased from American Type Culture Collection (ATCC, #CRL-3216)<br>THP1-NF-kB-Blue cells were purchased from Invivogen (#thp-nfkb)
Authentication	STR loci analysis was performed on all cell lines when received and after experimentation was complete.
Mycoplasma contamination	All cell lines are routinely tested and are confirmed to be negative for mycoplasma.
Commonly misidentified lines (See ICLAC register)	We did not use commonly misidentified lines.

Animals and other research organisms

Policy information about studies involving animals; ARRIVE guidelines recommended for reporting animal research, and Sex and Gender in Research

Laboratory animals	All mice were housed in the Association for Assessment and Accreditation of Laboratory Animal Care (AAALAC)–accredited animal facility at Cincinnati Children's Hospital Medical Center, maintained under specific pathogen-free (SPF) conditions and monitored under tightly controlled settings. They were housed on IVC racks (Allentown Jag 75 Micro-VENT Environmental Systems IVC racks, Allentown, NJ) and kept in individually ventilated polysulfone shoebox cages (Alternative Design, Siloam Springs AR), with up to four mice per cage. The cages were supplied with corncob bedding (Bed-o'Cobs ¼, The Andersons, Maumee OH), ad libitum feed (LabDiet 5010, St Louis MO), and enrichment (Twist-n'Rich, The Andersons, Maumee OH). All cage components were autoclaved before housing the mice, and cages were changed weekly. Mice had access to ad libitum water through a reverse osmosis autowater system. The mouse room was maintained on an automatic 12-hour light/dark cycle at an ambient temperature of 23°C, 30-70% humidity, and 5% Clidox-S was used as a disinfectant. Mice were bred, housed, and monitored daily by laboratory staff and veterinary personnel to ensure good health, activity, and the presence of appropriate food, water, and cage conditions. Quarterly testing of pathogens was conducted in sentinel animals housed in the same room. Excluded agents included: Mycoplasma pulmonis, CAR bacillus, Ectromelia, Rotavirus (EDIM), Hantaan virus, K virus, Lymphocytic choriomeningitis virus (LCMV), Mouse adenoviruses (MAV1, MAV2), Mouse cytomegalovirus (MCMV), Mouse hepatitis virus (MHV), Mouse parvovirus (MPV), Mouse thymic virus (MTV), Minute virus of mice (MVM), Polyoma virus, Pneumonia virus of mice (PVM), Reoviruses (REO3), Sendai virus, Theilers murine encephalomyelitis virus (TMEV), Encephalitozoon cuniculi, Aspiculuris tetraptera, Fur mites (Myocoptes, Radfordia/Myobia), and Pinworms (Aspiculuris tetraptera, Syphacia muris, Syphacia obvelata).<br>All the mice were maintained on C57BL/6J background. Dnmt3af/f and MxCre+ (obtained from H. Lee Grimes Laboratory, CCHMC), Dnmt3afl-R878H (B6(Cg)-Dnmt3atm1Trow/J, #032289, Jackson Laboratory), Tet2fl/fl (B6;129S-Tet2tm1.1Iaai/J, #017573, Jackson Laboratory), VavCre+ (B6.Cg-Commd10Tg(Vav1-icre)A2Kio/J, #008610, Jackson Laboratory), Alpk1-/- (11 bp deletion in exon 3, C57BL/6N-Alpk1em1Fsha/J, #032561, Jackson Laboratory), Tifa-/- (gift from Jun-Ichiro Inoue, University of Tokyo, Japan), and UBC-GFP (C57BL/6-Tg(UBC-GFP)30Scha/J, #004353, Jackson Laboratory). NF-kB-GFP reporter mice were generously provided by C. Jobin. Throughout the study, CD45.1+ B6.SJL- Ptprca (BoyJ) mice (6-10 weeks of age) were used as recipients for BM transplantation experiments. Littermate controls were used for all experiments. Mice at the age of 6-10 weeks and >52 weeks were chosen for young and old groups, respectively.
Wild animals	No wild animals were used in this study
Reporting on sex	Animals of both genders (male and female) were always included and randomly assigned to experimental groups
Field-collected samples	No field-collected samples were used in this study

Ethics oversight	All experiments were performed according to the animal guidelines upon approval of the Institutional Animal Care and Use Committee at CCHMC (IACUC2019-0072).

Note that full information on the approval of the study protocol must also be provided in the manuscript.

Flow Cytometry

Plots

Confirm that:

☒ The axis labels state the marker and fluorochrome used (e.g. CD4-FITC).

☒ The axis scales are clearly visible. Include numbers along axes only for bottom left plot of group (a 'group' is an analysis of identical markers).

☒ All plots are contour plots with outliers or pseudocolor plots.

☒ A numerical value for number of cells or percentage (with statistics) is provided.

Methodology

Sample preparation	Mice were euthanized using CO_2 followed by cervical dislocation. PB was collected into EDTA-coated tubes (Cat no. 22030403, Fisher Scientific), and hind limb bones (femurs, and tibias) were obtained immediately after euthanasia and stored in cold FACS buffer (1% FBS in DPBS) under sterile conditions. Bones were crushed using a mortar and pestle and then passed through a 40-µm cell strainer (Cat no. 542040, Greiner Bio-one). Primary bone marrow (BM) or peripheral blood (PB) cells were then filtered through a cell strainer (70 µm) to obtain a single-cell suspension. For analysis of hematopoietic cell surface markers, primary cells were washed once with 1X PBS (GE Healthcare Life Sciences), and subsequently stained with conjugated antibodies in FACS buffer (1% FBS in DPBS) for various applications.
Instrument	BD Fortessa, BD LSRII, FACSCanto, and SH800S (Sony Biotechnology).
Software	BD Facs Diva Software 8 and FlowJo 10.8
Cell population abundance	The frequency of sorted cell population was <0.1%. Sorted cells were re-analzyed to confirm the purity and it was >95%.
Gating strategy	In all experiments, debris were excluded by using Forward scatter/Side scatter (FSC/SSC). Doublets were excluded by double forward (FSC-A and FSC-W), and side scatter (SSC-A and SSC-H). Dead cells were excluded as DAPI+ cells. In the peripheral blood, donor cells were identified as CD45.2+ cells, and B cells, T cells, myeloid cells were gated as CD19+B220+, CD3+, Gr-1 +CD11b+ cells, respectively. In the bone marrow, HSCs were gated as Lin−Sca−1+c-Kit+Flk2-CD150+CD48− Positive gates were set with signal above 10^3.

☒ Tick this box to confirm that a figure exemplifying the gating strategy is provided in the Supplementary Information.
